# Electrically Controlled Metal‐Insulator Heterogeneous Evolution for Infrared Switch and Perfect Absorption

**DOI:** 10.1002/advs.202416420

**Published:** 2025-02-25

**Authors:** Xuefeng Cao, Jiahui Sun, Yuan Fang, Xurong Qiao, Shenghao Cai, Yuhao Qiu, Xuegang Chen, Yifei Sun, Jijie Huang, Xiangdong Ding, Jun Sun, Chenghao Wan, Zhen Zhang

**Affiliations:** ^1^ State Key Laboratory for Mechanical Behavior of Materials Xi'an Jiaotong University Xi'an 710049 China; ^2^ School of Materials Shenzhen Campus of Sun Yat‐sen University Shenzhen 518107 China; ^3^ Center of Free Electron Laser & High Magnetic Field Leibniz International Joint Research Center of Materials Sciences of Anhui Province Hefei 230601 China; ^4^ Information Materials and Intelligent Sensing Laboratory of Anhui Province Anhui Key Laboratory of Magnetic Functional Materials and Devices Anhui University Hefei 230601 China; ^5^ College of Energy Xiamen University Xiamen 3661005 China; ^6^ Department of Electrical Engineering Stanford University Stanford CA 94305 USA

**Keywords:** electrically controlled, infrared switch, metal‐insulator transition, perovskite nickelate, proton mediated

## Abstract

Active switching, which enables multifunctionality within a single optical component, is essential for reconfigurable infrared photonic systems such as radiation engineering, sensing, and communication. Metamaterials offer a solution but involve complex design and fabrication. A simpler approach with a planar layered structure becomes promising for offering economical manufacturing, easier integration, and scalability. However, it requires an active medium with giant tunability and effective modulation mechanisms. Here, an electrically controlled reversible infrared switching is demonstrated via a single layer of perovskite nickelate on an opaque substrate. Driven by the evolution of the refractive index during an electrically triggered proton‐mediated metal‐to‐insulator transition, the device transforms from a high reflective (*R* ≈0.74) to a low reflective state (*R* ≈0.09) at λ = 7–10 µm. A temperature‐independent perfect absorption (*A* > 0.99 at λ = 11.6–12.1 µm) emerges in the partially hydrogenated state with the mixture of the metal and insulator phases, which results in a modulation of emissivity ≈0.623 at λ = 7–14 µm. The switching behavior is tunable over a wide temperature and wavelength range, offering a versatile path for adaptive infrared applications.

## Graphical abstract

Electrically controlled optical switch and perfect absorption are achieved in the long wavelength infrared range by modulating the proton‐mediated metal‐insulator phase mixture of a single layer of SmNiO_3_ on an opaque sapphire substrate. Such an infrared switch is reversible and tunable over a broad temperature and wavelength range, which could shed light on the design of reconfigurable photonics.

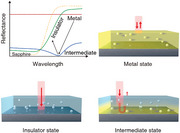

## 1 Introduction

Infrared light, though invisible to human eyes, plays a vital role in the survival of species and technological development. Organisms, such as rattlesnakes,^[^ ^1^ ^]^ cephalopods,^[^ ^2^, ^3^ ^]^ and Saharan silver ants,^[^ ^4^ ^]^ can hunt, camouflage, and regulate body temperature, respectively, via receiving and emitting infrared radiation.^[^ ^5^ ^]^ Inspired by these intriguing behaviors, infrared adaptive devices whose optical properties can be dynamically controlled according to environmental changes have been intensively explored and opened up new paths toward intelligent thermal management,^[^ ^6^, ^7^, ^8^, ^9^, ^10^, ^11^ ^]^ camouflage and imaging,^[^ ^12^, ^13^, ^14^ ^]^ and communication.^[^ ^15^, ^16^, ^17^, ^18^, ^19^ ^]^ A widely adopted paradigm for designing adaptive functionalities at the infrared wavelength range is based on the actively tunable metasurface, which is composed of subwavelength patterning of infrared active materials.^[^ ^15^, ^16^, ^17^, ^18^, ^19^ ^]^ This designing micro‐structure enables the metasurface devices to modulate the linear and circular polarization light via controlling the wavefront phase and Pancharatnam‐Berry phase respectively and exciting the electromagnetic resonances for both the amplitude and phase modulation in polarization independence cases.^[^ ^20^ ^]^ However, the complexity and high cost and time of designing and fabricating metasurface still challenge their large‐scale application.^[^ ^21^, ^22^ ^]^ Given manufacturing, scalability, and compatibility in building complex photonic systems, a planar layered architecture presents distinct advantages, including cost‐effective fabrication processes, enhanced integration capability, and improved scalability for implementation in diverse optical systems.

Achieving adaptive infrared functionality using a simple planar layered structure is challenging due to the requirement for the materials themselves to possess ultra‐high optical tunability. So far, electrochromic materials (WO_3_,^[^ ^23^, ^24^ ^]^ Li_4_Ti_5_O_12_^[^ ^25^ ^]^) dependent on redox reactions, phase‐change materials (VO_2_,^[^ ^26^, ^27^ ^]^ SmNiO_3_,^[^ ^19^, ^28^, ^29^ ^]^ Ge_2_Sb_2_Te_5_,^[^ ^30^ ^]^ (La,Sr)MnO_3_^[^ ^31^, ^32^ ^]^) based on metal‐insulator transition, conductive polymers materials^[^ ^33^, ^34^ ^]^ via controlling polaron generation and annihilation, and the graphene^[^ ^35^, ^36^ ^]^ with adjustable band structure and surface plasmon polaritons are developed (Table S1, Supporting Information). Among these active materials, the reconfigurable electronic ground states through transitions between metallic and insulating phases of strongly correlated materials provide a natural advantage in optical modulation.^[^ ^37^, ^38^, ^39^, ^40^, ^41^, ^42^ ^]^ The evolution of complex refractive index during the thermally induced metal‐insulator transition, such as observed in vanadium dioxide and perovskite nickelate, was found to enable unique functionalities close to the transition temperature (T_MIT_) at the infrared range, such as perfect absorption,^[^ ^38^ ^]^ zero‐differential thermal emitter,^[^ ^39^ ^]^ high sensitive thermography,^[^ ^43^ ^]^ and adaptive radiative cooling.^[^ ^9^, ^10^ ^]^ However, such intriguing functionalities are passively associated with temperature, which makes them transient during large thermal fluctuations. As a result, electronically compatible driving mechanisms of the phase evolution between metal and insulator would be promising to achieve active optical modulation with enhanced adaptability to dynamic complex environments.^[^ ^44^, ^45^, ^46^ ^]^ Instead of thermal excitations, electron‐filling‐induced metal‐to‐insulator transition through electrochemical hydrogenation has been found in several perovskite nickelates such as SmNiO_3_ and NdNiO_3_.^[^ ^47^, ^48^, ^49^ ^]^ Substantial changes in complex refractive index between pristine and fully hydrogenated were observed and utilized to design metasurfaces with tunable optical behaviors.^[^ ^19^ ^]^ However, the role of the metal‐insulator phase mixture on infrared optical behavior across the hydrogenation‐induced transition has not been systematically investigated. Whether thermally induced transient behavior of strongly correlated material, such as the perfect absorption in VO_2_,^[^ ^38^ ^]^ can emerge from the hydrogenation‐induced evolution between the metal and insulator phase is still unknown.

**Figure 1:**
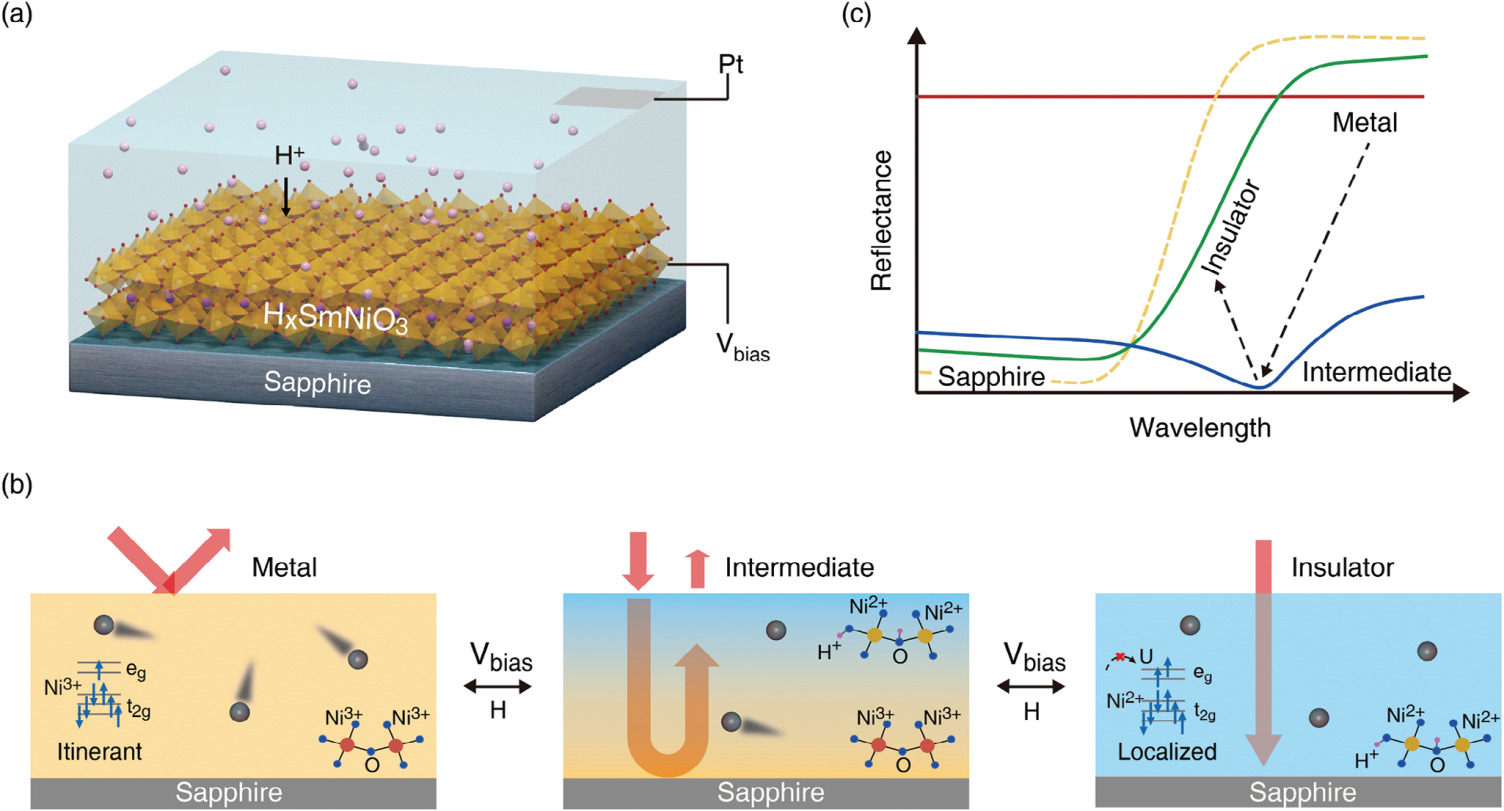
Schematic illustration of the electrically‐controlled perovskite nickelate long‐wavelength infrared switch. a) A bias voltage is applied to the SmNiO_3_ thin film deposited on an infrared opaque sapphire substrate across 0.01 m KOH solution. It triggers the interaction of proton into the SmNiO_3_ lattice, during which the microscopic evolution and optical behavior of the sample are depicted in b,c), respectively. Pristine SmNiO_3_ at the metallic state, featuring itinerant charge carriers, possesses high reflectance. Upon hydrogenation, the e_g_ orbital of Ni becomes half‐filling, which results in a metal‐to‐insulator transition and strong localization of charge carriers. At the intermediate state, where the metallic and insulating phases are mixed, the H_x_SmNiO_3_ film becomes a lossy dielectric layer and demonstrates perfect absorption with minimization of reflectance. When the SmNiO_3_ layer becomes fully hydrogenated, the thin film becomes insulating and transmissive in the long‐wavelength infrared range. It results in a low reflectance at a shorter wavelength, similar to the sapphire substrate. Since the proton can be de‐intercalated from SmNiO_3_ by reversing the bias voltage and thermal excitation, the evolution of the optical behavior of SmNiO_3_/sapphire is reversible.

In this work, we present an electrically controlled infrared switching and the emergence of temperature‐independent perfect absorption with a planar structured device composed of a hydrogenated perovskite nickelate on top of an opaque substrate. SmNiO_3_, as a prototypical strongly correlated material in the family of perovskite nickelate with a hysteresis‐free metal‐insulator transition at ≈130 °C,^[^ ^47^, ^48^, ^50^ ^]^ was grown on the sapphire substrate (**Figure** **1**a). Bias voltages were applied to the SmNiO_3_/sapphire device across 0.01 M KOH to trigger the proton intercalation. As a result, the SmNiO_3_ possessing the metallic state above T_MIT_ with itinerant carriers eventually transforms to a hydrogenated phase (HSmNiO_3_) (Figure 1b). Due to the half‐filled *e*_g_ orbital of HSmNiO_3_, strong Mott‐Hubbard electron‐electron interaction arises and drives the materials into an insulating state with localized carriers. As Figure 1c shows, the device transforms from a high reflective to a low reflective state in the shorter wavelength region (7–10 µm) upon such a transition, which is attributed to the increase of transmittance of SmNiO_3_ upon hydrogenation with the low reflectance of the opaque sapphire substrate. In the intermediate state, where the metallic and insulating phases coexist, the reflectance of the device can approach zero locally (i.e., close to perfect absorption) by meeting the critical coupling condition in the 10–14 µm wavelength region (Figure 1b,c). As a result, a substantial modulation on emissivity of ≈0.623 can be achieved between the metallic and partially hydrogenated states. These distinct optical states of SmNiO_3_/sapphire can be switched reversibly by applying a reverse bias voltage and thermal excitation. By substituting Sm with Nd and controlling the thickness of the SmNiO_3_ layer, the infrared switching behavior of SmNiO_3_/sapphire can be tuned over broad temperature and wavelength ranges. Our work suggests a new route toward reconfigurable photonic devices based on the electrically controlled phase evolution of correlated materials.

## 2 Results and Discussion

### 2.1 Reversible Infrared Switching Behavior of SmNiO_3_/Sapphire

SmNiO_3_ film was synthesized on the sapphire substrate in this work via the chemical solution deposition method. X‐ray diffraction profile of pristine SmNiO_3_/sapphire device confirms its orthorhombic structure (Figure S1a, Supporting Information). Cross‐section and top‐view Scanning Electron Microscopy images (Figure S1b,c, Supporting Information) demonstrate that the SmNiO_3_ film prepared here is polycrystalline and uniform with a thickness of ≈210 nm. The temperature‐dependent reflection spectra of pristine SmNiO_3_/sapphire (Figure S2, Supporting Information) reveal the existence of thermally triggered metal‐insulator transition. The transition temperature T_MIT_ is ≈135 °C with negligible hysteresis, consistent with previous works.^[^ ^51^, ^52^ ^]^ The experimental setup to switch the long wavelength infrared behavior of SmNiO_3_/sapphire is depicted in Figure S3 (Supporting Information). The electrically controlled switching was carried out electrochemically using a standard three‐terminal potentiostat in 0.01 M KOH (Figure S3a, Supporting Information) with SmNiO_3_/sapphire device working as a working electrode. Bias voltages (v.s. Ag/AgCl) were applied to SmNiO_3_/sapphire to trigger the intercalation of proton and the onset of hydrogenation‐mediated metal‐insulator transition. A fully hydrogenated SmNiO_3_/sapphire sample, taken as a reference, was prepared by annealing in H_2_ gas at elevated temperature (Figure S3b, Supporting Information).

**Figure 2:**
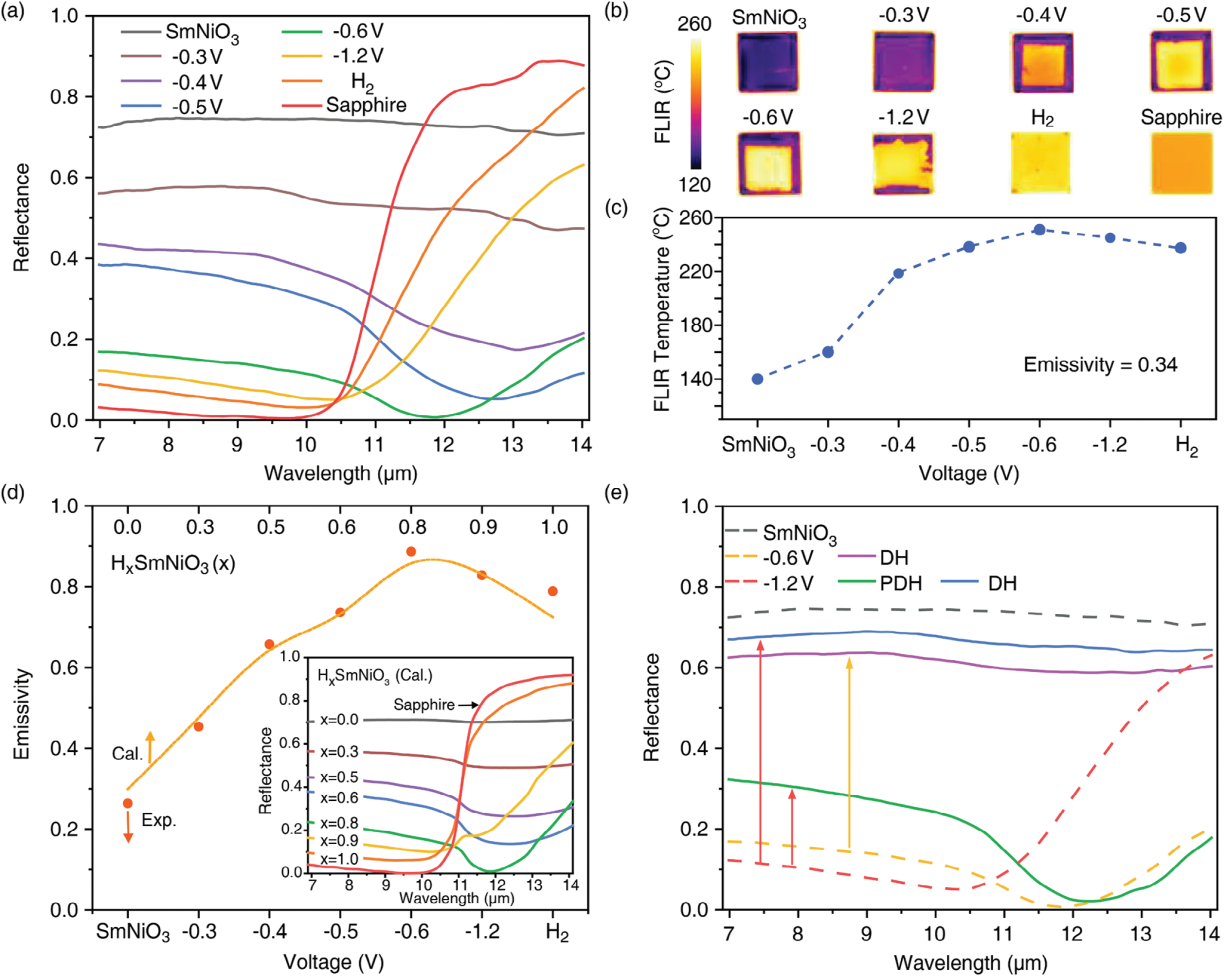
Electrically driven evolution on optical properties of SmNiO_3_/sapphire in the wavelength range of 7–14 µm. a) Reflectance of the sample after application of bias voltage. The Fourier Transform Infrared (FTIR) spectroscopy measurement was conducted at 140 °C to stabilize the metallic state of pristine SmNiO_3_. A fully hydrogenated sample was obtained by annealing in H_2_ gas. The reflectance of the sample decreases from ≈0.74 to ≈0.09 in the wavelength of 7–10 µm. At the intermediate state (−0.6 V), the sample shows perfect absorption with *R* < 0.01 at λ = 11.6–12.1 µm. b) Long wavelength thermal images and c) apparent temperature of the sample during switching measured by a FLIR camera. A substantial modulation of emissivity can be observed. d) Emissivity of the sample as a function of bias voltage, the value of which was deduced from its reflection spectra. An increase of emissivity Δ ≈0.623 appears. The inset shows the calculated reflectance of the H_x_SmNiO_3_/sapphire device, where the switching behavior is reproduced. The experimentally observed emissivity (Exp.) can be fitted well with the calculated one (Cal.). e) Reflectance of the sample upon partial and complete de‐hydrogenation (PDH and DH, respectively). The reflectance of the sample can recover from hydrogenation at both −0.6 and −1.2 V, indicating the reversibility of SmNiO_3_/sapphire upon switching.

The evolution of the reflectance of SmNiO_3_/sapphire at 140 °C in the wavelength range of 7–14 µm under bias voltages is shown in **Figure** **2**a. Since the measurement temperature is above T_MIT_, the metallic state is thermally stabilized in pristine SmNiO_3_. As a result, the SmNiO_3_/sapphire without bias voltage applied shows a high reflectance of *R* ≈0.74 in λ = 7–14 µm. With increasing the bias voltage, the reflectance of the sample gradually decreases. At the bias voltage of −0.6 V, the reflectance of the sample reaches a minimum value of ≈0.0068 at λ = 11.8 µm, being ≈6X lower than the reflectance minimum observed in the thermally stabilized insulation state of the pristine sample (0.0402 at λ = 13.4 µm, see Figure S2a, Supporting Information). Since the sapphire substrate is opaque in λ = 7–14 µm, the sample transforms to an absorption‐dominated state with *A* > 0.99 at the broad wavelength of λ = 11.6–12.1 µm. With further increasing the bias voltage, the sample shows low reflectance with *R *≈0.09 at λ = 7–10 µm. Its reflection spectrum becomes similar to that of the sapphire substrate, indicating that the SmNiO_3_ thin film becomes transparent at high bias voltage.

The evolution of the reflectance of SmNiO_3_/sapphire under the application of bias voltage was further illustrated by long‐wavelength thermal imaging using an infrared camera (Figure 2b,c). The bias voltage was selectively applied in the center area of the film, which appears as the rectangle region in the thermal images. With the increment of bias voltage, the apparent temperature of the sample increases gradually and reaches a maximum at the bias voltage of −0.6 V, beyond which the apparent temperature reduces slightly and approaches that of the bare sapphire substrate. The modulation in emissivity of the sample during switching was further deduced from its reflectance based on Kirchhoff's law (Note S1, Supporting Information). As shown in Figure 2d, the emissivity of the sample increases first and then drops with enlarging the bias voltage, consistent with the thermal images. A maximum modulation of emissivity (Δε ≈0.623) is achieved between the pristine and intermediate states. A summary of switchable optical devices based on infrared active materials present here and previous reports is shown in Table S1 (Supporting Information). It can be found that the infrared emissivity modulation of the active infrared device based on hydrogenated SmNiO_3_ shown here is higher than that made from representative electrochromic material WO_3_ (Δε: 0.2–0.4)^[^ ^23^, ^24^ ^]^ and close to prototypical thermochromic material VO_2_ (Δε: 0.4–0.6)^[^ ^26^, ^27^ ^]^ and (La,Sr)MnO_3_(Δε: 0.4–0.65)^[^ ^31^, ^32^ ^]^ as well as phase change material Ge_2_Sb_2_Te_5_ (Δε ≈0.62).^[^ ^30^ ^]^

To reveal the infrared switching behavior of SmNiO_3_/sapphire under bias voltage, the reflectance of the devices with the H_x_SmNiO_3_ layer on the sapphire substrate was further calculated (Note S2, Supporting Information). Based on Lichtenecker's mixing rule, the complex refractive indices of H_x_SmNiO_3_ films at different hydrogenation levels were obtained by changing the ratio of SmNiO_3_ and HSmNiO_3_. With increasing the level of hydrogenation, a monotonic reduction on both parts of the complex refractive index occurs (Figure S4, Supporting Information). At the pristine state, the SmNiO_3_ transfers to the metallic state above T_MIT_ where its imaginary part of refractive index *k* increases at longer wavelength (Figure S4b, Supporting Information) indicating its Drude‐like behavior. As a result, the pristine SmNiO_3_/sapphire shows a high calculated reflectance of ≈0.71 in λ = 7–14 µm (Figure 2d inset), consistent with experiment observations (*R* ≈0.74). When the SmNiO_3_ is fully hydrogenated, the imaginary part of the complex refractive index approaches zero in 7–14 µm Figure S4b, Supporting Information). As a result, the HSmNiO_3_ layer becomes transparent. The device shows a low calculated reflectance of *R* ≈0.07 in λ = 7–10 µm, similar to the bare sapphire substrate (Figure 2d inset).

In the intermediate state where SmNiO_3_ and HSmNiO_3_ are mixed, the value of the imaginary and real part of the complex refractive index becomes close (Figure S4, Supporting Information), implying the lossy dielectric nature of the H_x_SmNiO_3_. Based on the calculated reflection spectra as a function of *x* (Figure 2d inset), such a heterogeneous state of H_x_SmNiO_3_ on the sapphire substrate enables a crossover to the critical coupling condition. When *x* = 0.8, the incident infrared light becomes almost fully absorbed (*R*_min_ = 0.0078) at a wavelength of ≈11.9 µm. Such a calculated result coincides with experimental observations (i.e., *R*_min_ = 0.0068 at λ = 11.8 µm) when SmNiO_3_/sapphire is hydrogenated under a bias voltage of −0.6 V. The reproduction of experimental results (Figure 2a) with the calculation ones (Figure 2d inset) implies that the electrically controlled switching of SmNiO_3_/sapphire results from a substantial modulation on the complex refractive index of the SmNiO_3_ thin film upon proton intercalation under bias voltage.

Moreover, we found that the switching behavior of SmNiO_3_/sapphire is reversible upon de‐hydrogenation (DH, see method section). As Figure 2e shows, the intermediate state achieved by the applied voltage of −0.6 V can be recovered back to the reflective state after DH. After applying a high bias voltage of −1.2 V for hydrogenation, the sample can be switched back to the intermediate state after partial de‐hydrogenation (PDH) by applying a reverse bias of +1.2 V for 135 s. A further transition to the high reflectance state can be achieved by combining a longer duration of the reverse bias voltage at +1.2 V for 30 min with additional annealing in oxygen gas at an elevated temperature of 300 °C. Thermal images confirm the evolution of emissivity during the switching and recovering process (Figure S5, Supporting Information). After the DH treatment, the thermally induced metal‐insulator transition recovers (Figure S6, Supporting Information). In addition, negligible degradation in the morphology of SmNiO_3_ is observed after the DH process (Figure S7, Supporting Information).

The long‐term stability of the SmNiO_3_/sapphire device upon electrically controlled switching was further studied. We measured the reflectance of the sample in the reflective and perfect absorptive states at every 20 switching cycles. The switching between the two states was conducted at −1 V and +1 V respectively in 0.01 M KOH for 1 min. As shown in Figure S8 (Supporting Information), the reflectance and modulation on the emissivity of the SmNiO_3_/sapphire device remain similar over 140 switching cycles, which demonstrates its stability upon switching.

We further study the role of film thickness on the infrared switching behavior of the SmNiO_3_/sapphire device. The reflection spectra of devices with the thickness of SmNiO_3_ layer spanning from 50 to 300 nm were calculated (Figure S9, Supporting Information). It is found that the switching behavior of SmNiO_3_/sapphire persists with varying film thickness. The reflectance of devices in pristine and fully hydrogenated states at λ = 7–10 µm increases with larger film thickness. Moreover, the spectral location of the perfect absorption at the intermediate state shifts continuously to longer wavelengths with increasing film thickness. As a result, the modulation of emissivity during switching increases gradually with film thickness and shows a maximum at thickness of ≈250 nm (Figure S10, Supporting Information). This indicates that engineering the thickness of the SmNiO_3_ layer is an effective way to tune the switching behavior of the device.

### 2.2 Mechanism on the Infrared Switching Behavior of SmNiO_3_/Sapphire

**Figure 3:**
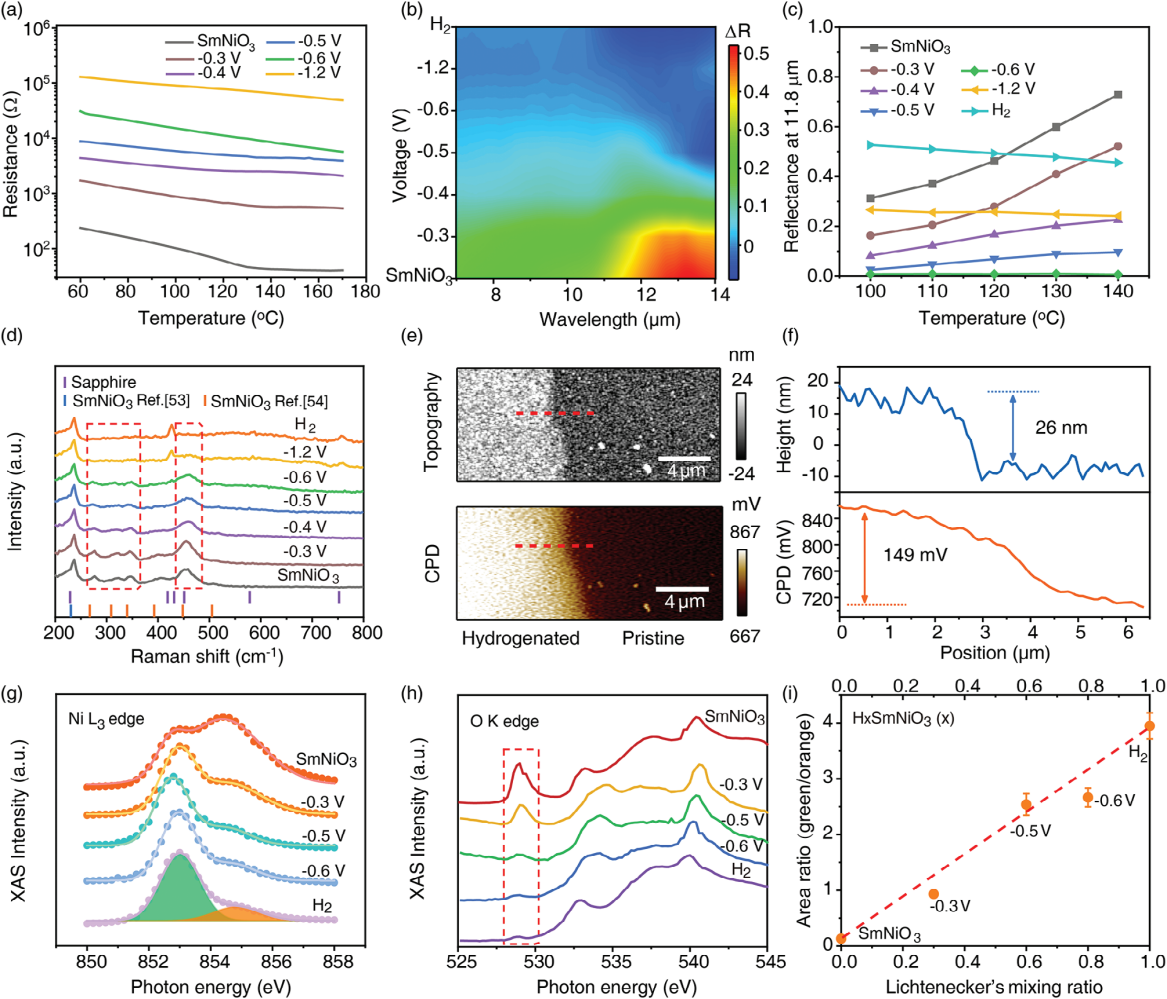
Mechanism of electrically controlled modulation of SmNiO_3_/sapphire infrared switch. a) Temperature‐dependent electrical resistance of SmNiO_3_ after application of bias voltage. The resistance increases by ≈3 orders of magnitude, indicating the onset of the Mott transition in SmNiO_3_. b) Change in reflectance (Δ*R*) of the sample between 110 and 140 °C. The Δ*R* becomes approximately zero above – 0.6 V, indicating a decoupling of reflectance with temperature. c) Temperature‐dependent reflectance of the sample at λ = 11.8 µm. A temperature‐invariant perfect absorption is achieved at −0.6 V. d) Raman spectra of the sample during switching. Typical Raman modes^[^ ^53^, ^54^ ^]^ of SmNiO_3_ are diminished with increasing bias voltage. e) Atomic force microscopy topography and KPFM contact potential difference (CPD) of a selective‐area hydrogenated SmNiO_3_. The hydrogenated area was obtained by applying a bias voltage of −0.6 V for 5 min. f) Height and CPD profile along the red dashed line in (e). After hydrogenation, an expansion of film thickness by ≈12.4% and a reduction of CPD by ≈149 mV occur. g) Ni L_3_ edge and h) O K edge X‐ray absorption spectra of SmNiO_3_ during switching. i) Linear correlation between the peak area ratio of the Ni L_3_ edge and the Lichtenecker's mixing ratio of the insulating phase in H_x_SmNiO_3_ during switching.

To explore the infrared switching behavior of SmNiO_3_/sapphire, we carried out temperature‐dependent electrical resistance measurements to investigate the transition features of the films upon application of bias voltage **Figure** **3**a. It can be found that with increasing bias voltage, the electrical resistance of samples exhibits a substantial increment by ≈3 orders of magnitude, indicating the occurrence of electrically driven proton‐mediated metal‐insulator transition.^[^ ^47^ ^]^ Moreover, the thermally induced metal‐insulator transition becomes absent above −0.6 V, suggesting that modulation of the temperature‐dependent reflectance of samples could occur.

Subsequently, we systematically measured the evolution of the reflectance of the samples spanning from 140 to 100 °C (Figure S11, Supporting Information). The difference in reflectance between 140 and 100 °C is further depicted in Figure 3b. It can be found that the reflectance of the HSmNiO_3_/sapphire with applied bias voltage above −0.6 V becomes decoupled from temperature variation. As a result, the reflectance of the absorption‐dominated intermediate state obtained at a bias voltage of −0.6 V becomes invariant upon temperature fluctuations (Figure 3c). The sample demonstrates an ultralow reflectance of ≈0.0068 at λ = 11.8 µm spanning from 100 to 140 °C.

Raman spectra (Figure 3d) of samples were investigated upon the voltage‐induced switching.^[^ ^53^, ^54^ ^]^ The Raman mode of SmNiO_3_ within 250–370 cm^−1^ gradually diminishes with increasing bias voltage and vanishes in the fully hydrogenated state (i.e., −1.2 V and H_2_‐treated ones). Simultaneously, the Raman mode near 450 cm^−1^shifts to higher values with increasing bias voltage. Such a modification of Raman spectra is consistent with previous literature reports implying a modification of the crystal structure of SmNiO_3_ involving rearrangement of NiO6 octahedron during electrically triggered hydrogenation.^[^ ^55^, ^56^ ^]^ Moreover, the Raman modes of the sapphire substrate at 586 and 758 cm^−1^ become visible at the voltage of −1.2 V, confirming the enhancement of transmission as the sample approaches the fully hydrogenated state.

The proton‐mediated metal‐insulator transition of SmNiO_3_ thin film under electrical bias is further probed microscopically using the Kelvin probe force microscopy (KPFM) (Figure 3e,f). Comparing the contact potential difference (CPD) and topography between the hydrogenated and pristine region of the film, an expansion of film thickness of ≈12.4% occurred with the introduction of proton into the lattice. Moreover, an increase of CPD (i.e., reduction of work function Φ_*sample*_ = Φ_*tip*_  − *e* · *CPD*) by ≈149 mV is observed at the hydrogenated region, indicating the occurrence of electron filling accompanied by the proton intercalation.

To estimate the level of hydrogenation in SmNiO_3_ quantitatively upon infrared switching, the X‐ray absorption near edge structures of Ni L_3_ and O K edges were carried out. Figure 3g,h shows the X‐ray absorption spectra (XAS) of SmNiO_3_ upon applied bias voltage of −0.3, −0.5 , and −0.6 V. A fully hydrogenated SmNiO_3_ via H_2_ annealing was also studied as a reference. It can be found that the spectrum weight in Ni L_3_ peak shifts from higher energy at ≈854.5 eV to lower energy at ≈853 eV. Simultaneously, the pre‐edge peak at O K‐edge (≈529 eV) becomes suppressed. Such a modification of XAS spectra suggests an electron filling into the unoccupied states of Ni‐O hybridized orbitals during hydrogenation.^[^ ^47^ ^]^ As a result, the XAS spectrum of Ni L_3_ edge for fully hydrogenated SmNiO_3_ becomes similar to that of NiO, where the valence state of Ni changes into 2+.^[^ ^57^, ^58^ ^]^ Due to the half‐filling at e_g_ orbitals of Ni^2+^ in 3d^8^ state (Figure 1b), a strong Mott‐Hubbard correlation among electrons arises, which triggers a filling‐induced metal‐insulation transition.^[^ ^59^ ^]^ By taking the area ratio through a double Gaussian fitting of the Ni L_3_ XAS peak and comparing it with Lichtenecker's mixing ratio used in optical calculations, a linear correlation between these two sets of values is observed in Figure 3i. As a result, such anti‐doping‐induced evolution on the peak profile of the Ni L_3_ edge enables a quantitative method to obtain the mixing ratio of Ni^2+^ and Ni^3+^ as well as the level of hydrogen in the H_x_SmNiO_3_. Moreover, it confirms the crucial role of a heterogeneous mixture of metallic phase with Ni^3+^ and insulating phase with Ni^2+^ in hydrogenated SmNiO_3_ in determining the evolution of their optical response at the long wavelength infrared regime.

### 2.3 Manipulation on Temperature Window of the Infrared Switching

The temperature‐dependent reflection spectra of pristine SmNiO_3_/sapphire show that its reflection‐dominated state appears at the thermally stabilized metallic phase above T_MIT_ (≈135 °C)_._ Therefore, to exploit the infrared switching behavior of SmNiO_3_/sapphire at lower temperatures, a method to reduce the T_MIT_ of SmNiO_3_ is required. It is reported that substituting Sm with other rare‐earth elements, which modulates its Goldschmidt tolerance factor $t=\left(r_{A}+r_{O}\right)/\sqrt{2}\left(r_{B}+r_{O}\right)$, can effectively regulate the T_MIT_ of SmNiO_3_.^[^ ^60^, ^61^ ^]^ The *r*_A_, *r*_B_, and *r*_O_ represent the ionic radii of rare‐earth elements, Ni, and O ions. To reduce the T_MIT_, therefore, lanthanide elements with a larger ionic radius than Sm are expected. As a result, Nd, Pr, and La become promising candidates. Among these lanthanide elements, the difference in ionic radius between Nd and Sm is the smallest, indicating high solubility. Hence, to gradually reduce the T_MIT_ without introducing secondary phases, Nd was chosen to substitute Sm for this work. Nd_x_Sm_1‐x_NiO_3_ films with x = 0.15, 0.25, 0.35, and 0.4 were synthesized. As shown in Figure S12 (Supporting Information), Nd_x_Sm_1‐x_NiO_3_ samples possess the perovskite structure with no impurities. The substitutional Nd element is uniformly distributed. Moreover, the Raman modes of films (Figure S12b, Supporting Information) shift continuously toward NdNiO_3_^[^ ^62^ ^]^ with increasing the Nd concentration, indicating the successful substitution of Sm with Nd in present samples.

**Figure 4:**
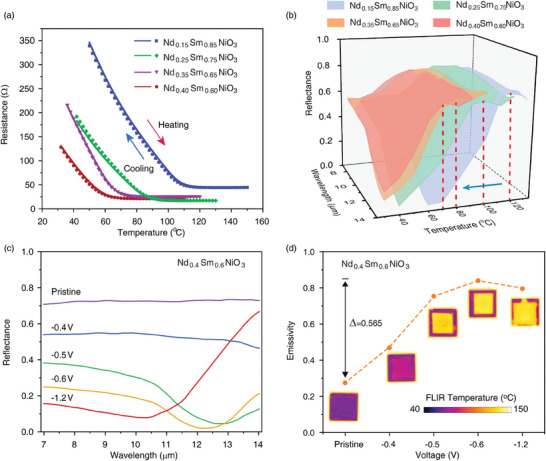
Manipulation on the working temperature of SmNiO_3_/sapphire infrared switch. a) Temperature‐dependent electrical resistance of Nd_x_Sm_1‐x_NiO_3_. The onset temperature of the thermally induced metal‐insulator transition decreases gradually with Nd substitution. b) Reflectance of Nd_x_Sm_1‐x_NiO_3_ upon heating. With Nd substitution, the temperature to obtain the reflection‐dominated pristine state decreases monotonically. c) Evolution on the reflectance of Nd_0.4_Sm_0.6_NiO_3_/sapphire as a function of bias voltage at 70 °C. Switching behavior similar to SmNiO_3_/sapphire is observed. d) Emissivity and long wavelength thermal image of Nd_0.4_Sm_0.6_NiO_3_/sapphire as a function of bias voltage. Upon Nd substitution, the device can retain a substantial modulation of emissivity (≈0.565).

As revealed by the temperature‐dependent electrical resistance of Nd_x_Sm_1‐x_NiO_3_ (**Figure** **4**a), the T_MIT_ reduces monotonically from ≈135 °C to 114, 94, 76, and 66 °C with x = 0.15, 0.25, 0.35, and 0.4, respectively. Moreover, the electrical resistance upon cooling (dot) and heating (solid line) overlays with each other, indicating the free of thermal hysteresis. The reflectance of Nd_x_Sm_1‐x_NiO_3_ upon heating obtained from temperature‐dependent Micro‐FTIR measurement is shown in Figure 4b. The reflection‐dominated state in the 7–14 µm wavelength range, coupling with its T_MIT_, is shifted gradually to a lower temperature regime in the pristine Nd_x_Sm_1‐x_NiO_3_/sapphire samples.

To verify the existence of proton‐mediated switching behavior in Nd_x_Sm_1‐x_NiO_3_ films on the sapphire substrate, we carried out the same hydrogenation process for Nd_0.4_Sm_0.6_NiO_3_ as that for SmNiO_3_ by applying bias voltage. The reflectance of Nd_0.4_Sm_0.6_NiO_3_/sapphire devices after hydrogenation at various bias voltages was measured at 70 °C and shown in Figure 4c. Similar switching behaviors are reproduced in H_x_Nd_0.4_Sm_0.6_NiO_3_/sapphire with increasing the bias voltage. The device transforms from the high reflective pristine state to the low reflective state in λ = 7–10 µm at the bias voltage of −1.2 V and approaches perfect absorption in λ ≈12.3 µm at the intermediate state (−0.6 V). The emissivity deduced from the reflectance and the corresponding thermal images (Figure 4d) further confirm the switching behavior of H_x_Nd_0.4_Sm_0.6_NiO_3_/sapphire samples. The maximum modulation of the emissivity during switching can still reach a high value of 0.565. Therefore, our results demonstrate that the perovskite nickelate infrared switch is applicable across a broad temperature range.

## 3 Conclusion

In conclusion, our work demonstrates that giant reversible optical modulation can be achieved in the long wavelength infrared range by driving the metal‐to‐insulator transition of a single layer of strongly correlated nickelate perovskite on an opaque substrate through electrically controlled hydrogenation from metallic state. Moreover, the heterogeneous mixture of metallic and insulating phases at the intermediate state enables a temperature‐independent perfect absorption due to approaching the critical coupling condition. The electrically controlled phase transition of correlated material, coupled with substantial modulation of optical behavior, paves the way toward reconfigurable photonic devices with simple and scalable structures.

## 4 Experimental Section

### SmNiO_3_ Film Synthesis

SmNiO_3_ films were synthesized on sapphire substrates by chemical solution deposition methods. The 0.2 M SmNiO_3_ precursor solution was prepared by Sm(NO_3_)_3_·6H_2_O (Sigma‐Aldrich) and Ni(CH_3_COO)_2_·4H_2_O (Sigma‐Aldrich), which were dissolved in 2‐methoxyethanol (Sigma‐Aldrich) at 1:1 stoichiometric ratio. Before spin‐coating, the sapphire substrate was cleaned using acetone, isopropanol, and deionized water, followed by an oxygen plasma treatment to increase the surface wettability with the solution. A 50 µl precursor solution was dropped on the sapphire substrate and held for 10, 5, 5, and 20 s at 500, 3000, 3500, and 5000 rpm, respectively. The sample was baked at 180 and 340 °C for 10 min to evaporate the organic solvent and enhance adhesion. To obtain films with a thickness of ≈210 nm in this work, the spin‐coating and baking processes were repeated 8 times. The deposited films were then annealed at 340 °C for 30 min and subsequently at 800 °C for 12 h under 12 MPa pure oxygen gas to form the perovskite phase.

### Electrically Controlled Hydrogenation and De‐Hydrogenation of SmNiO_3_

The incorporation of hydrogen in the SmNiO_3_ films was carried out electrochemically in 0.01 M KOH with a potentiostat. A three‐terminal cell configuration was utilized with Pt as the counter electrode, SmNiO_3_ film as the working electrode, and Ag/AgCl as the reference electrode. Bias voltages of −0.3, −0.4, −0.5, −0.6, and −1.2 V (vs Ag/AgCl) were applied for 5, 5, 5, 5, and 100 min, respectively, to control the concentration of hydrogen in SmNiO_3_.

To obtain a fully hydrogenated SmNiO_3_ as a reference, nanosized Pd particles acting as the catalyst for hydrogenation were deposited on top of a sample by magnetron sputtering. The Pd deposition was conducted at a power of 50 W for 8 s.^[^ ^63^ ^]^ Then, the sample was hydrogenated at an elevated temperature of 100 °C in 3% H_2_ for 100 min.

PDH was conducted electrochemically at a bias voltage of +1.2 V (vs Ag/AgCl) for 135 s. To reach a full DH, the sample was electrochemically treated at a bias voltage of +1.2 V (vs Ag/AgCl) for 30 min, followed by high‐temperature annealing for 60 min in pure oxygen gas at 300 °C.

### Optical Measurements

The temperature‐dependent infrared reflectance of the sample was measured by Bruker Vertex 70 FTIR and Hyperion 3000 microscope with a reflective objective of 0.4 numerical aperture. The samples were kept at each temperature step using a temperature‐controlled stage for 1 min before the FTIR measurement. The standard gold mirror was used as a reference for reflectance measurement.

FLIR A615 camera, sensitive to the 7.5 to 14 µm range, was used for thermal imaging. To reveal the evolution of optical properties of SmNiO_3_/sapphire during switching, the emissivity parameter of the FLIR camera was set to match the apparent temperature of the pristine sample with its actual temperature.

### Electrical Measurements

The temperature‐dependent electrical resistance of samples was measured by the two‐point probes method using Keithley 2635B source meter. The heating and cooling rate was controlled at 5 °C min^−1^ in this work.

### Raman Spectroscopy

The micro‐Raman spectra were measured by the LabRam HR Evolution spectrometer. The laser with a wavelength of 532 nm was excited by the Nd: YAG laser. The numerical aperture of the microscope objective was 0.5, and the spot diameter was ≈1.3 µm. The Raman spectra were integrated 60 s twice at the power of 4 mW for each measurement.

### X‐ray Absorption Spectroscopy

The XAS measurement was carried out at beamlines MCD‐A and MCD‐B (Soochow beamline for energy materials) at the National Synchrotron Radiation Laboratory (NSRL, China). The XAS spectra of Ni L edge and O K edge were measured by the total electron yield at high vacuum (≈10^−5^ mbar). The spot size of the X‐ray was 1.5 mm × 2 mm.

### X‐ray Diffraction

The Rigaku SmartLab with two Ge(220) monochromator and Cu *K*_α_ (λ = 1.5406Å) was used to perform the XRD measurement of Nd_x_Sm_1‐x_NiO_3_ (x = 0, 0.15, 0.25, 0.35, and 0.4) and sapphire substrate. The increment step and hold time of measurement were 0.01° and 0.075 s, respectively.

### Scanning Electron Microscopy

The Sigma 300 FE‐SEM (ZEISS) and Ultim Max 100 (Oxford Instruments) were used to characterize the morphology and energy dispersive spectroscopy (EDS) of samples. The acceleration voltages during morphology and EDS measurements were set as 10 and 15 kV, respectively.

### Kelvin Probe Force Microscopy

The KPFM measurements were performed using Oxford Instruments Asylum Research Cypher ES environmental atomic force microscopy system with a Pt‐coated tip made by MikroMasch. The film was grounded during the measurement. The scanning area was 20 µm × 20 µm.

## Conflict of Interest

The authors declare no conflict of interest.

## Supporting information



Supporting Information

## Data Availability

The data that support the findings of this study are available from the corresponding author upon reasonable request.
