# Supplementary material for: Electrically Controlled Metal‐Insulator Heterogeneous Evolution for Infrared Switch and Perfect Absorption
Source: Adv Sci (Weinh). 2025 Feb 25;12(16):2416420. doi: 10.1002/advs.202416420 (PMC12021118; doi:10.1002/advs.202416420)
Supplement: Supplementary file 1 — Supporting Information [file ADVS-12-2416420-s001.docx]

Supporting Information

Electrically Controlled Metal-Insulator Heterogeneous Evolution for Infrared Switch and Perfect Absorption

*Xuefeng Cao, Jiahui Sun, Yuan Fang, Xurong Qiao, Jijie Huang, Shenghao Cai, Yuhao Qiu, Xuegang Chen, Yifei Sun, Xiangdong Ding, Jun Sun, Chenghao Wan, Zhen Zhang^*^*

1. **Supplementary note for emissivity calculations**

Since the sapphire substrate is opaque within the long wavelength infrared regime, the spectral emissivity of the samples can be calculated by reflectance measurement based on Kirchhoff’s law as follows:^[1]^

$\varepsilon_{S}\left( \lambda,T \right)=A\left( \lambda,T \right)=1-R(\lambda,T)$ (1)

where *A* is absorption and *R* is reflectance.

Further, the integrated emissivity can be calculated by the following formula:^[2]^

$\varepsilon\left( T \right)=\frac{\int_{\lambda_{1}}^{\lambda_{2}} \varepsilon_{s}(\lambda,T)I_{BB}(\lambda,T)}{\int_{\lambda_{1}}^{\lambda_{2}} I_{BB}(\lambda,T)}$ (2)

where *I_BB_(λ, T)* is spectral radiance of blackbody.

1. **Supplementary note for optical calculations**

Considering the multiple reflections of light waves in the single layer of H_x_SmNiO_3_ film on the sapphire substrate, the total reflection coefficient *r* of the sample should be the vector sum of the whole reflection coefficient. The analytical solution of *r* can be calculated as follows:^[3]^

$r=\frac{r_{12}+r_{23}e^{2i\beta}}{1+r_{12}r_{23}e^{2i\beta}}$ (3)

$r_{ij}=\frac{(\tilde{n}_{i}-\tilde{n}_{j})}{(\tilde{n}_{i}+\tilde{n}_{j})}$ (4)

$\beta=\frac{2\pi}{\lambda_{0}}\tilde{n}_{2}h$ (5)

$R=\left| r \right|^{2}$ (6)

where *r_ij_* is the Fresnel reflection coefficient (normal incidence) from medium i to j, and $\tilde{n}_{i}$ is the complex refractive index of medium i. The refractive index of the sapphire substrate was extracted from the reference.^[4]^ *λ*_0_ is the wavelength of incident light. *h* is the thickness of the H_x_SmNiO_3_ film. The reflectance *R* of the sample is the absolute square of *r*.

Since both SmNiO_3_ and HSmNiO_3_ are non-magnetic within the measurement temperature window of this study, the complex refractive index of H_x_SmNiO_3_ at various hydrogenation levels was estimated by Lichtenecker’s mixing rule as follows:^[5]^

$\tilde{\varepsilon}_{mix}^{k}=\left( 1-f \right)\tilde{\varepsilon}_{1}^{k}+f\tilde{\varepsilon}_{2}^{k}$ (7)

$\tilde{n}=\sqrt{\tilde{\varepsilon}}$ (8)

where $\tilde{\varepsilon}_{1}$ and $\tilde{\varepsilon}_{2}$ are the complex dielectric functions of SmNiO_3_ and HSmNiO_3,_ which were obtained from previous literature.^[1, 6]^ *f* represents the fraction of phase 2 (the HSmNiO_3_ here) in H_x_SmNiO_3_. The exponent *k* was taken as 1/3 here to characterize the anisotropy of H_x_SmNiO_3_ during the proton-mediated metal-insulator transition.^[5]^


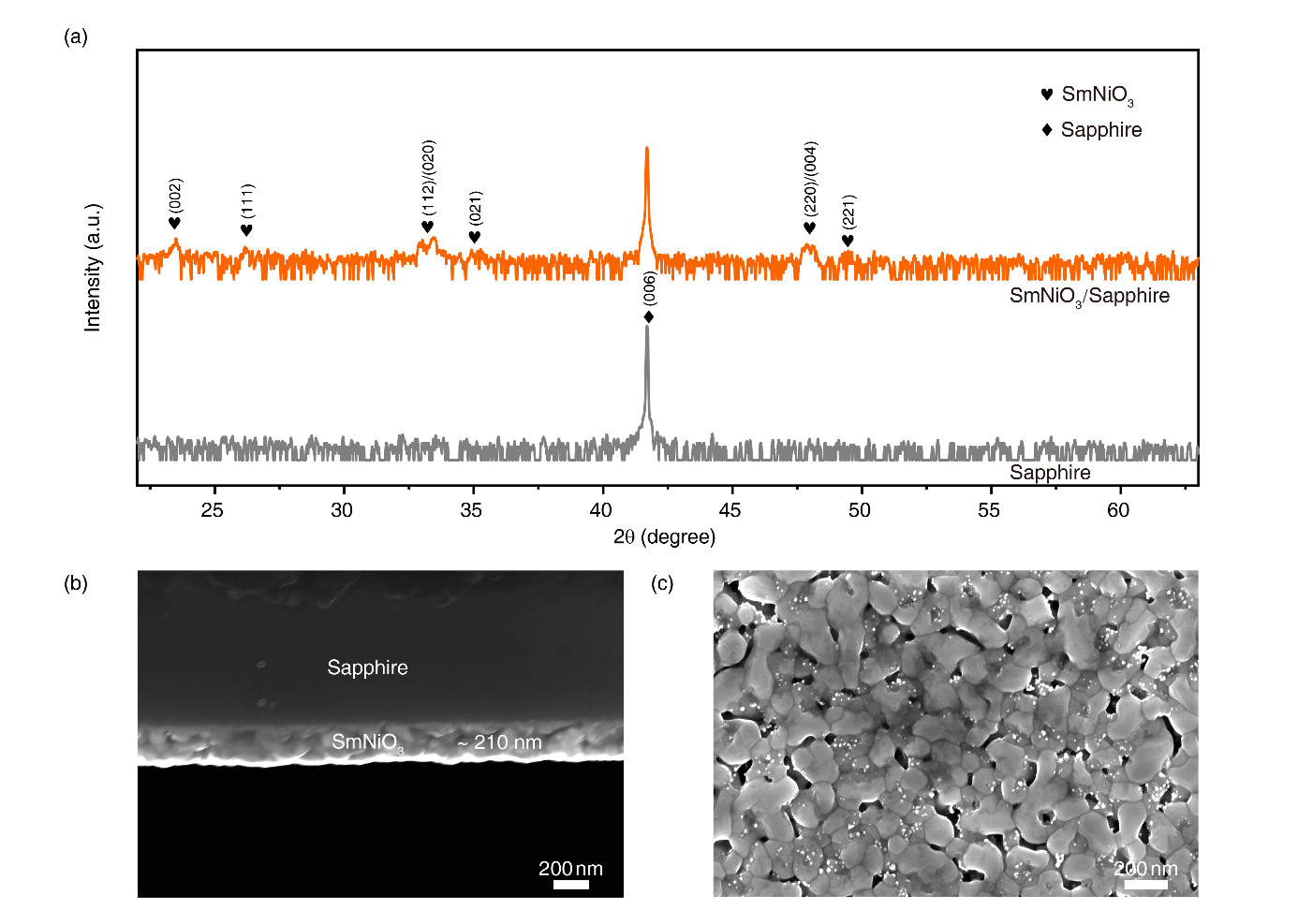


**Figure S1.** Structural characterization of pristine SmNiO_3_/sapphire device. a) Wide range XRD profile of the sample. Diffraction peaks from various crystal planes of SmNiO_3_ are observed, indicating its polycrystalline nature. b) Cross section and c) Top view SEM images of the sample. The thickness of the SmNiO_3_ layer is about 210 nm. The SmNiO_3_ shows a porous structure due to the chemical solution deposition method employed in this study. The pore size is approximately 55 nm, much shorter than the wavelength of infrared light (7-14 μm). As a result, these pores are not detectable in the FTIR spectrum.


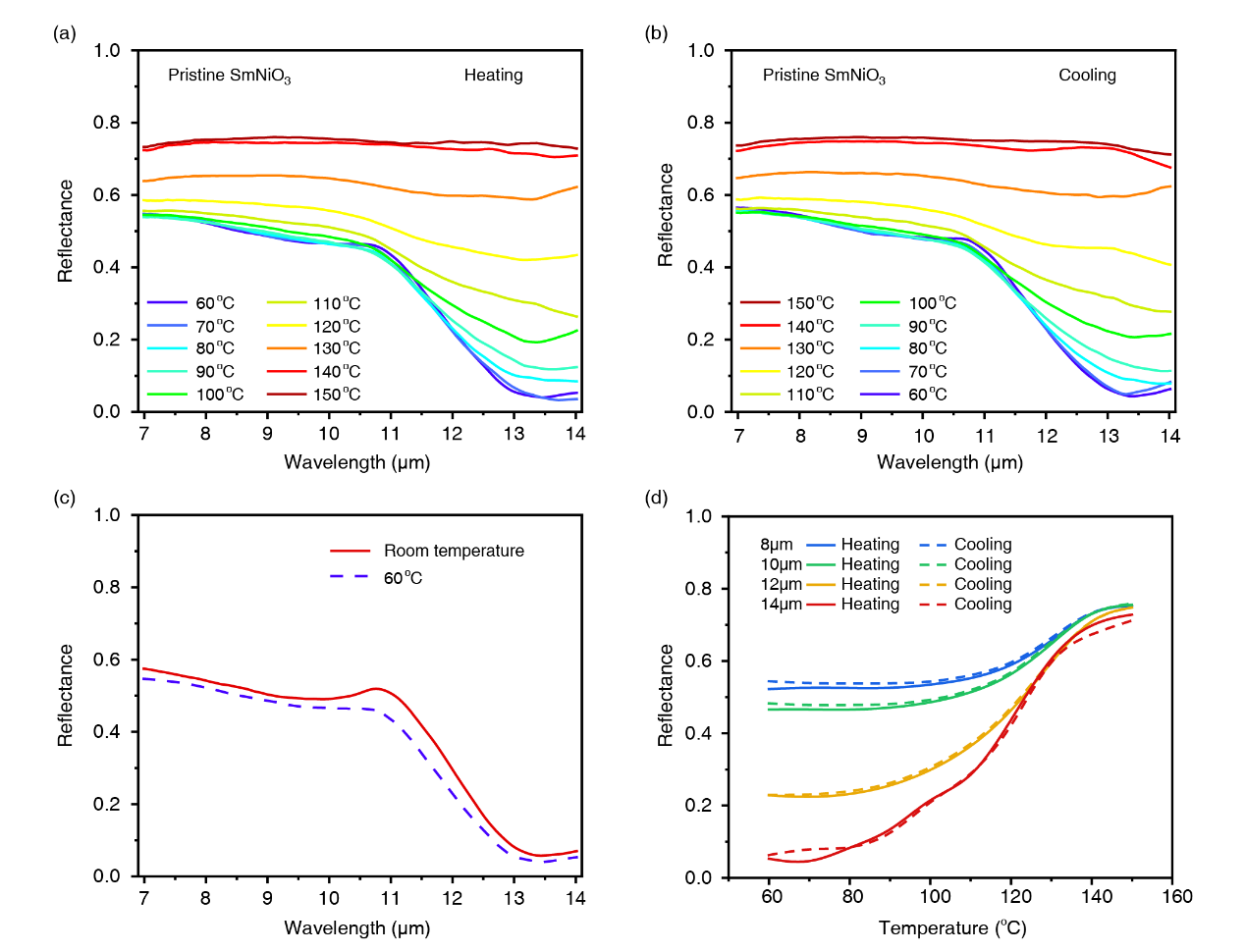


**Figure S2.** Temperature-dependent reflection spectra of SmNiO_3_/sapphire infrared switch at pristine state. A substantial evolution of the reflectance can be found during a) heating and b) cooling. The sample shows a high reflectance of ~0.74 above 140 ^o^C, indicating the stabilization of the metal phase at such a temperature range. Upon cooling, the reflectance of the sample decreases gradually due to the onset of thermally induced metal-to-insulator transition. The reflectance of the sample at 13.43 μm reduces to ~ 0.05 at 60 ^o^C and keeps a similar value down to the room temperature c). d) Temperature-dependent reflectance of the sample at the wavelength of 8, 10, 12, and 14 μm. The transition temperature T_MIT_ of SmNiO_3_ synthesized in this work is ~ 135 ^o^C. Moreover, the reflectance–temperature curves during cooling and heating overlap with negligible hysteresis, which is consistent with the gradual phase-change feature of the thermally induced metal-to-insulator transition in SmNiO_3_ thin films reported previously.^[7]^


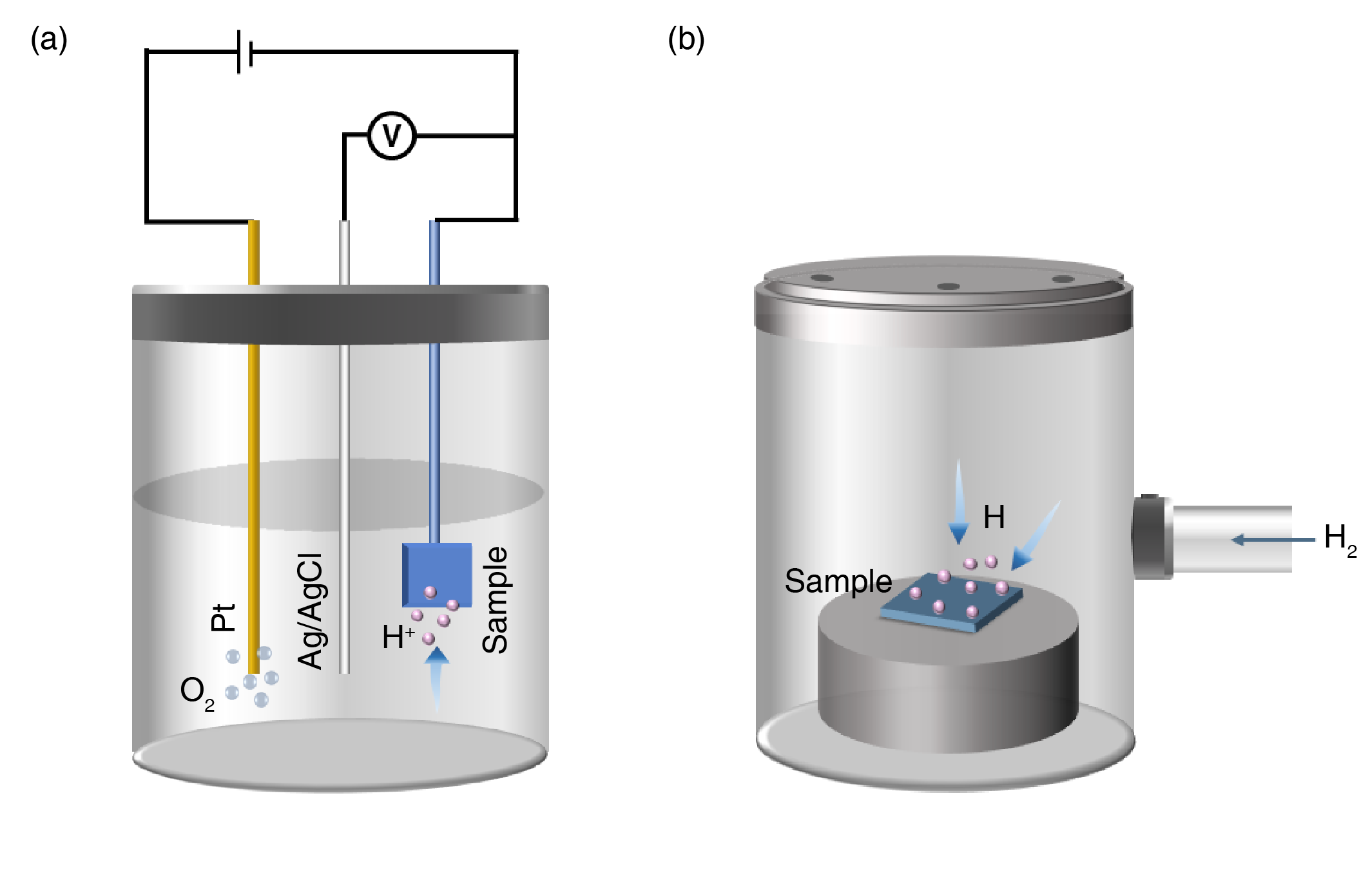


**Figure S3.** Method to modulate the infrared optical properties of SmNiO_3_/sapphire devices. a) Schematic illustration of the setup to switch the infrared optical properties of the sample by using electrical bias. The electrically controlled switch was conducted using a standard three-terminal potentiostat in 0.01 M KOH. The SmNiO_3_/sapphire device acted as a working electrode. Pt and standard Ag/AgCl were used as counter and reference electrodes. A bias voltage (vs. Ag/AgCl) was applied to the SmNiO_3_/sapphire device to enable the hydrogenation-mediated Mott transition and drive the evolution of their infrared behaviors. b) Schematic of catalyst-assisted hydrogenation. Pd nanoparticles, acting as a catalyst for hydrogenation, were deposited on the surface of a SmNiO_3_/sapphire sample by magnetron sputtering. Then, hydrogenation of the Pd/SmNiO_3_/sapphire device was achieved by annealing at an elevated temperature in H_2_ gas. In this work, we approached the fully hydrogenated state of SmNiO_3_/sapphire device by annealing the sample at 100 ^o^C in 3% H_2_ for 100 min.


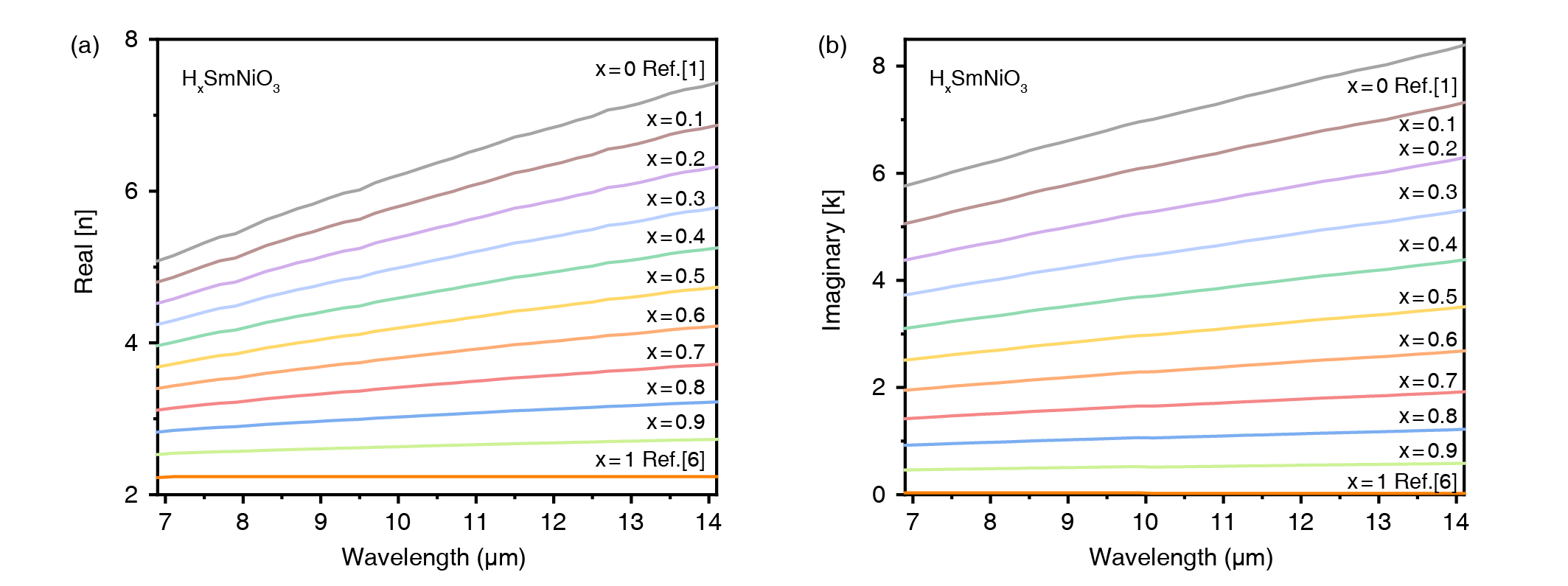


**Figure S4.** a) Real and b) imaginary parts of the complex refractive index of H_x_SmNiO_3_ at 140 ^o^C. In this work, the complex refractive index of H_x_SmNiO_3_ was estimated by Lichtenecker’s mixing rule (Supplementary Note 2). The exponent *k*, which is used to characterize the anisotropy of the H_x_SmNiO_3_ upon the proton mediated metal to insulator transition, was taken as 1/3.^[5]^ The complex refractive index of pristine SmNiO_3_ and fully hydrogenated HSmNiO_3_ at 140 ^o^C were extracted from references.^[1, 6]^


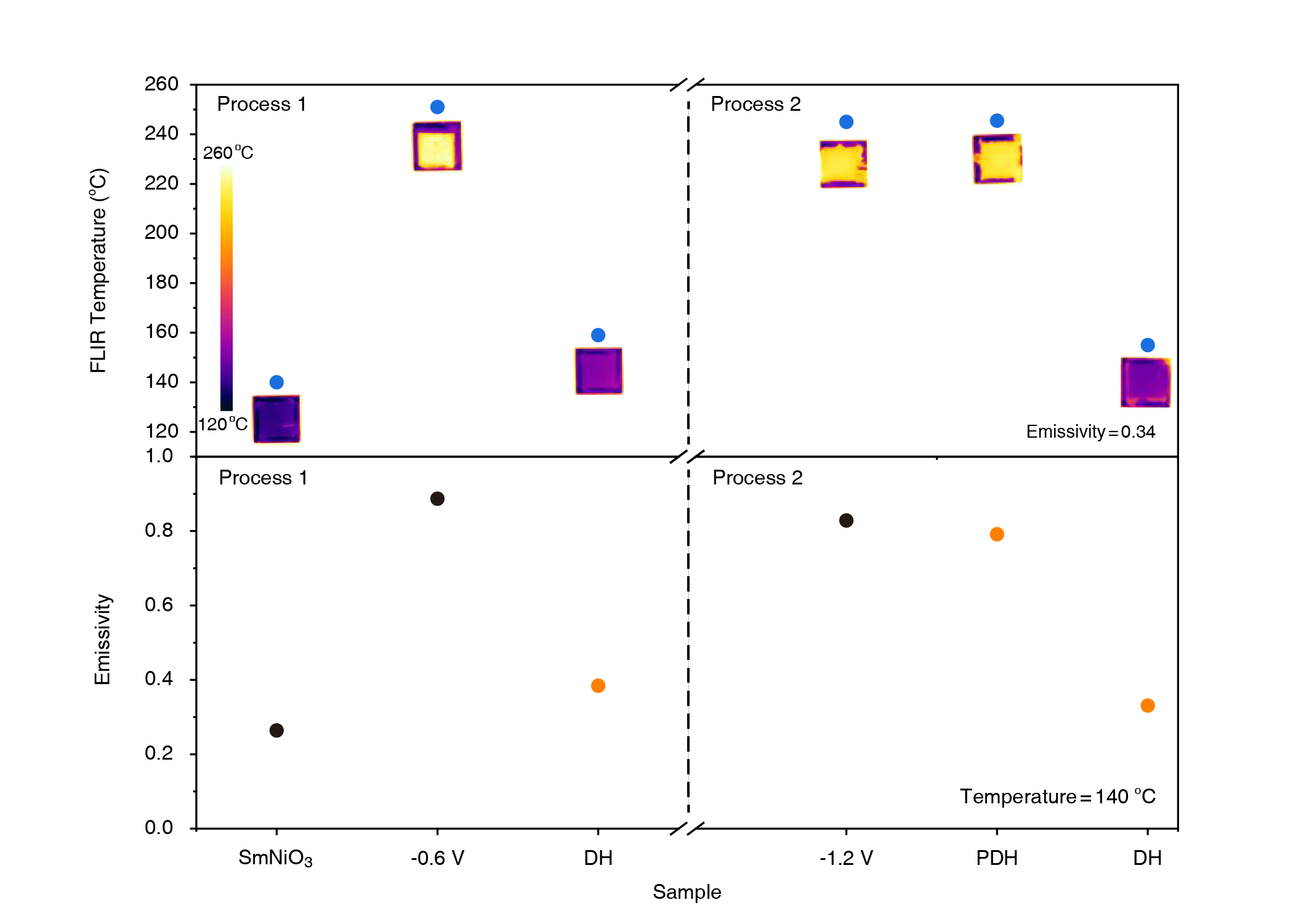


**Figure S5.** Thermal images of SmNiO_3_/sapphire upon de-hydrogenation. The upper panel shows the evolution of the apparent temperature and thermal images of the sample during the process. An emissivity of 0.34 was set in the FLIR camera during the measurement, the value of which was selected to minimize the difference between the apparent and actual temperature of the sample at the pristine state. The lower panel shows the emissivity of the sample, which was calculated from its reflectance in Figure 2e. In process 1, the pristine sample was hydrogenated under -0.6 V and then de-hydrogenated (DH) under +1.2 V for 30 min, followed by pure oxygen annealing. After DH, the sample showed low emissivity, close to the pristine state. In process 2, the pristine sample was intensively hydrogenated under the -1.2 V, followed by applying a voltage of +1.2 V for 135 s to achieve partial de-hydrogenation (PHD). Then, full de-hydrogenation was achieved by applying +1.2 V for 30 min, followed by pure oxygen annealing. During PHD, the sample transformed from the insulating to the intermediate state. After full de-hydrogenation (DH), the sample eventually recovered back to the low emissive state**.**


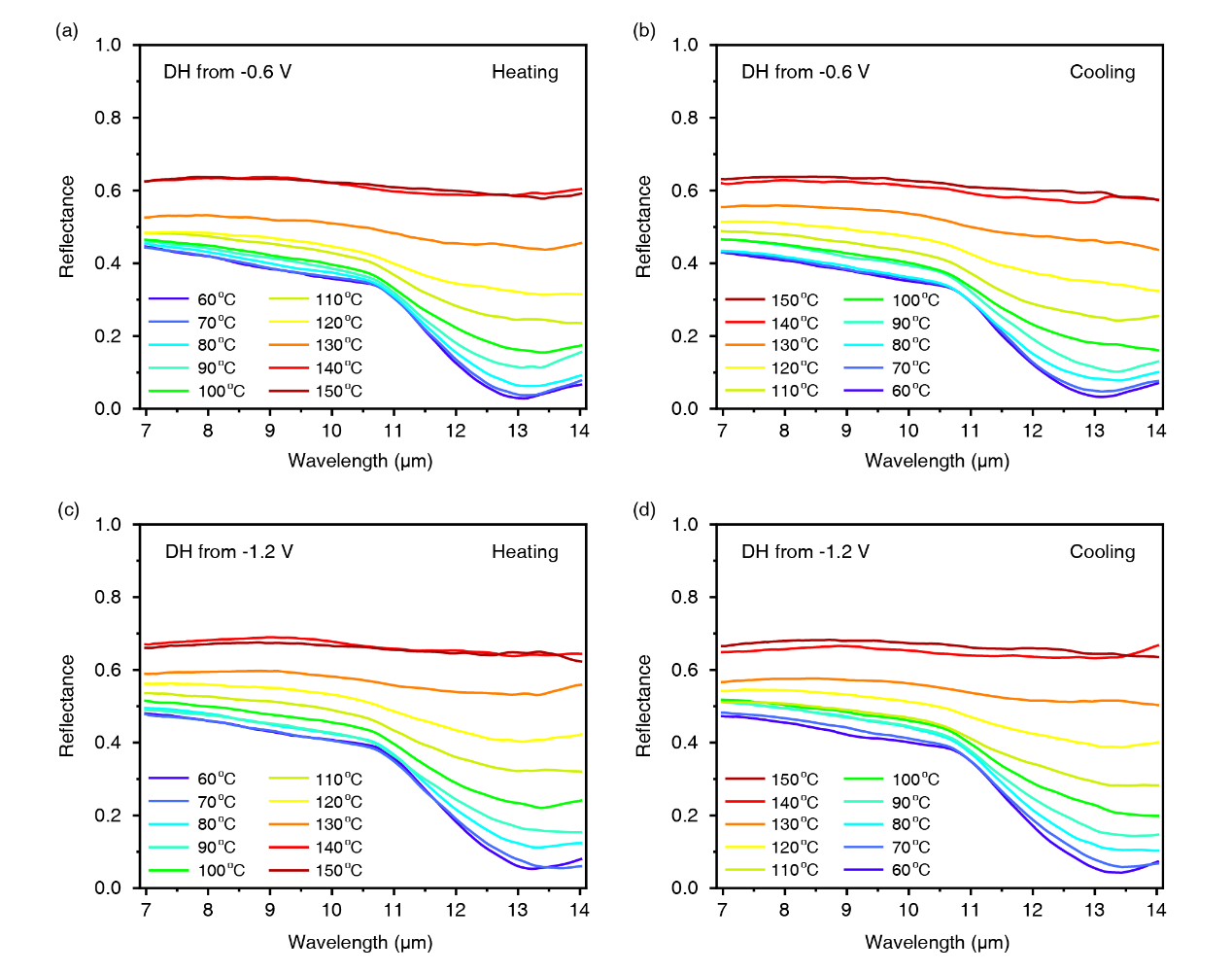


**Figure S6.** Temperature-dependent reflection spectra of de-hydrogenated SmNiO_3_/sapphire infrared switch. a) and b) Reflectance of the de-hydrogenated (DH) device upon heating and cooling, respectively. The bias voltage applied for hydrogenation was -0.6 V. After applying a higher voltage of -1.2 V during hydrogenation, the reflection spectra of the de-hydrogenated SmNiO_3_/sapphire sample during heating and cooling are shown in c) and d). It can be found that both samples possess temperature-dependent evolution of reflectance similar to that of pristine SmNiO_3_/sapphire device (Figure S2).


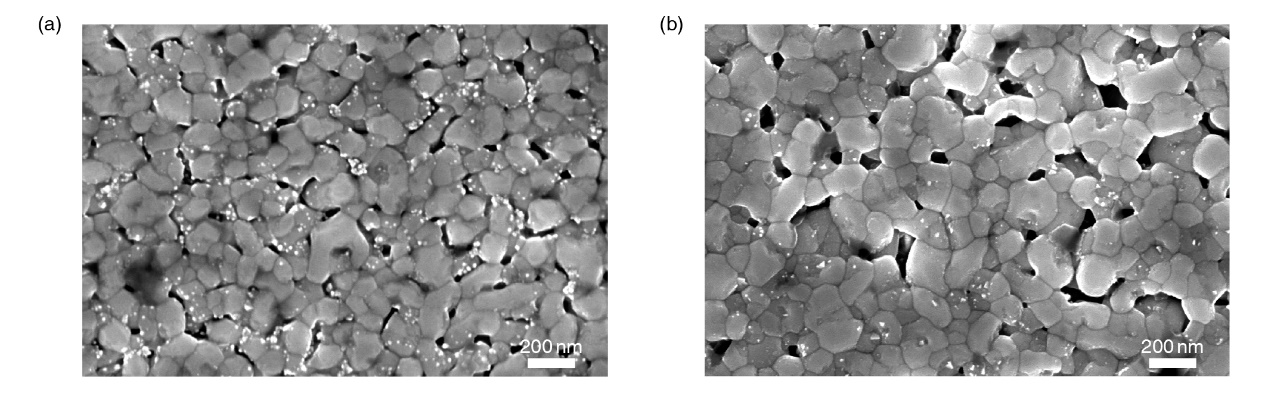


**Figure S7.** SEM images of SmNiO_3_/sapphire infrared switch upon electrically driven a) hydrogenation and b) de-hydrogenation. A voltage of -1.2 V was applied during the hydrogenation. The morphology of the SmNiO_3_ layer remains similar upon hydrogenation and de-hydrogenation, indicating the stability of SmNiO_3_ upon switching.


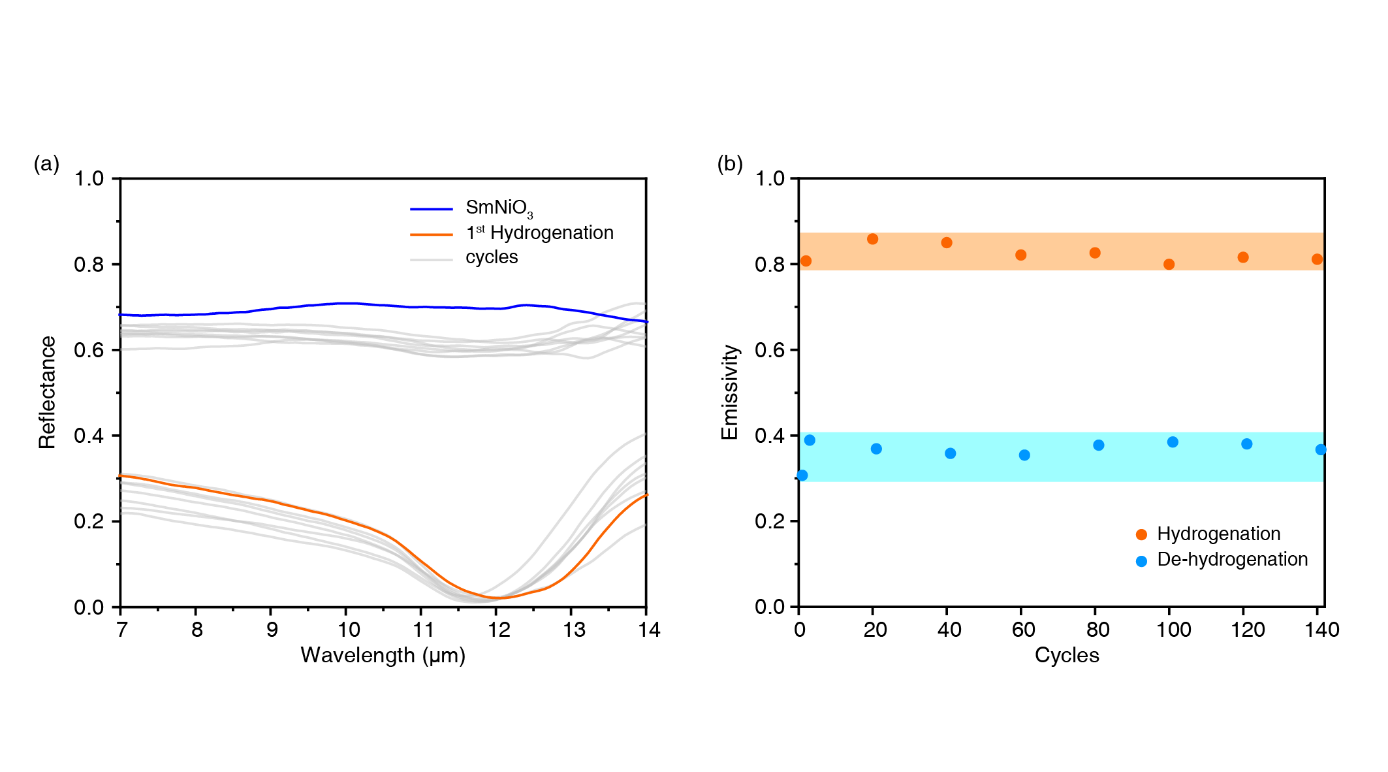


**Figure S8.** Stability of SmNiO_3_/sapphire device upon switching between reflective and perfect absorption states. a) Reflection spectra of SmNiO_3_/sapphire during switching over 140 cycles. During switching, the hydrogenation and de-hydrogenation were conducted at -1 V and +1 V in 0.01 M KOH for 1 min. b) Emissivity of the SmNiO_3_/sapphire device during switching.


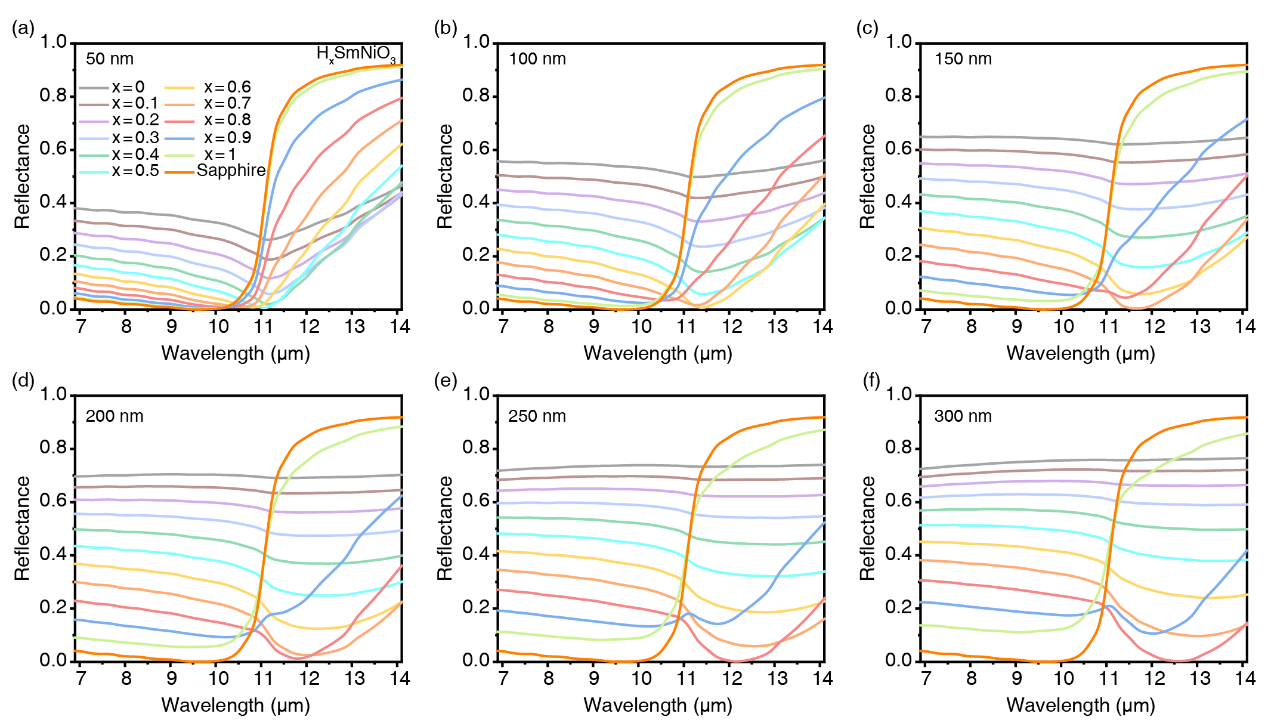


**Figure S9.** Calculated reflectance of SmNiO_3_/sapphire devices upon switching. The thickness of the SmNiO_3_ thin film was set as a) 50 nm, b) 100 nm, c) 150 nm, d) 200 nm, e) 250 nm, and f) 300 nm, respectively. All the samples demonstrate the infrared switching behavior upon hydrogenation. The reflectance of the SmNiO_3_/sapphire device at the pristine state becomes enhanced with increasing the film thickness. In addition, by controlling the thickness of SmNiO_3_, the spectral location where perfect absorption occurs can be modulated in the wavelength range of 11~13 μm.


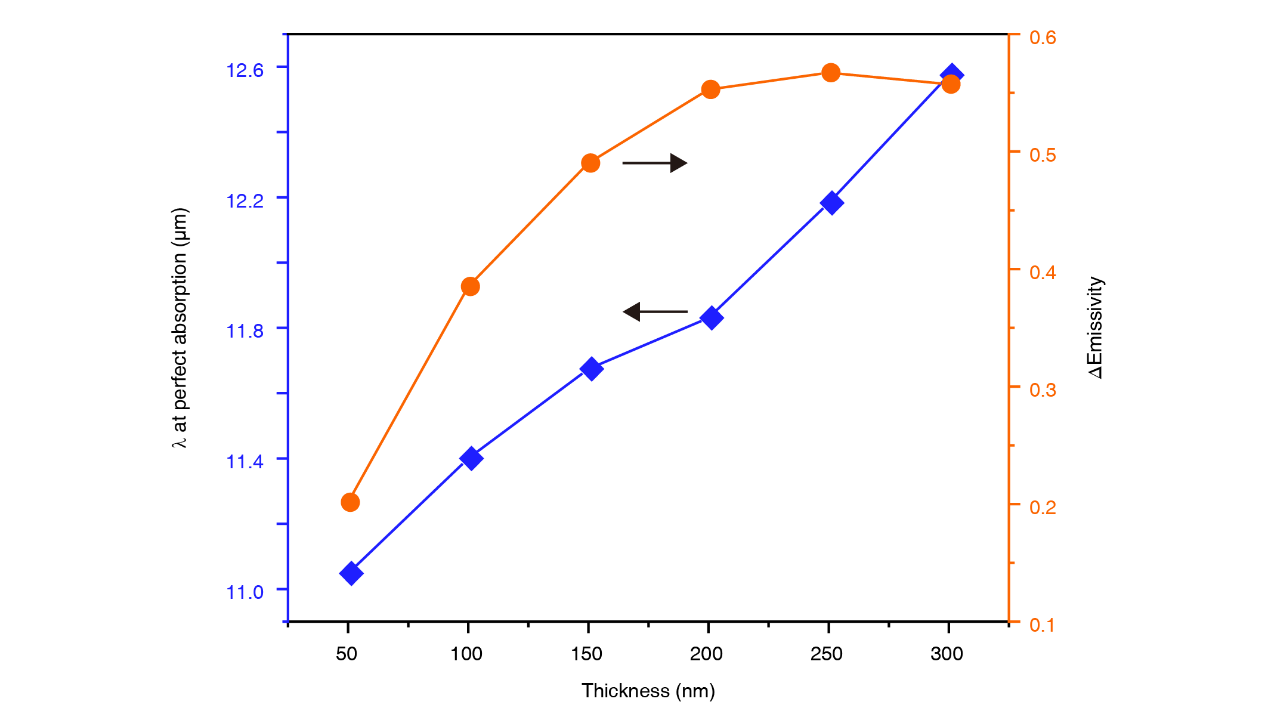


**Figure S10.** Spectral location of the perfect absorption and emissivity modulation of SmNiO_3_/sapphire infrared switch as a function of SmNiO_3_ layer thickness. The spectral location where perfect absorption occurs at the intermediate state shifts continuously to a longer wavelength upon increasing the thickness of SmNiO_3_. Simultaneously, the calculated modulation of infrared emissivity during switching increases from ~ 0.2 to ~ 0.55. As a result, a wide range of tuning on the infrared switching behavior of SmNiO_3_/sapphire device can be achieved by engineering the film thickness of SmNiO_3_.


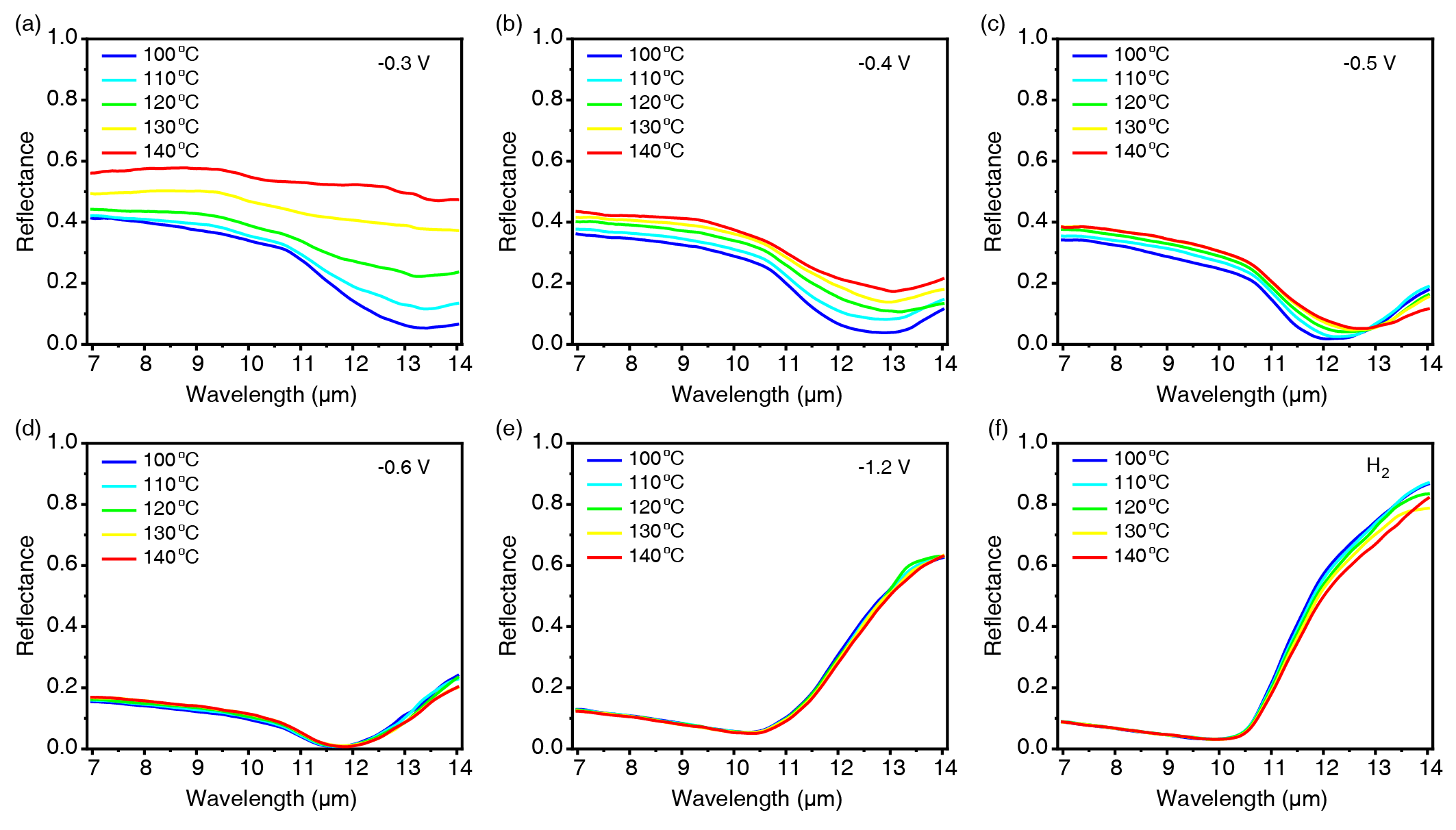


**Figure S11.** Temperature-dependent reflection spectra of SmNiO_3_/sapphire infrared switch measured after bias voltages were applied. The bias voltage was set as a) -0.3 V, b) -0.4 V, c) -0.5 V, d) -0.6 V, and e) -1.2 V, respectively. f) Reflectance of a H_x_SmNiO_3_/sapphire sample with *x* approaches 1. This sample was obtained by annealing in H_2_ gas at 100 ^o^C for 100 min. When the driving voltage is higher than -0.6 V, the reflectance of the device becomes almost independent of temperature. It is consistent with the gradual suppression of the thermally induced metal-insulator transition in H_x_SmNiO_3_, as observed in their electrical resistance–temperature curves (Figure 3a).


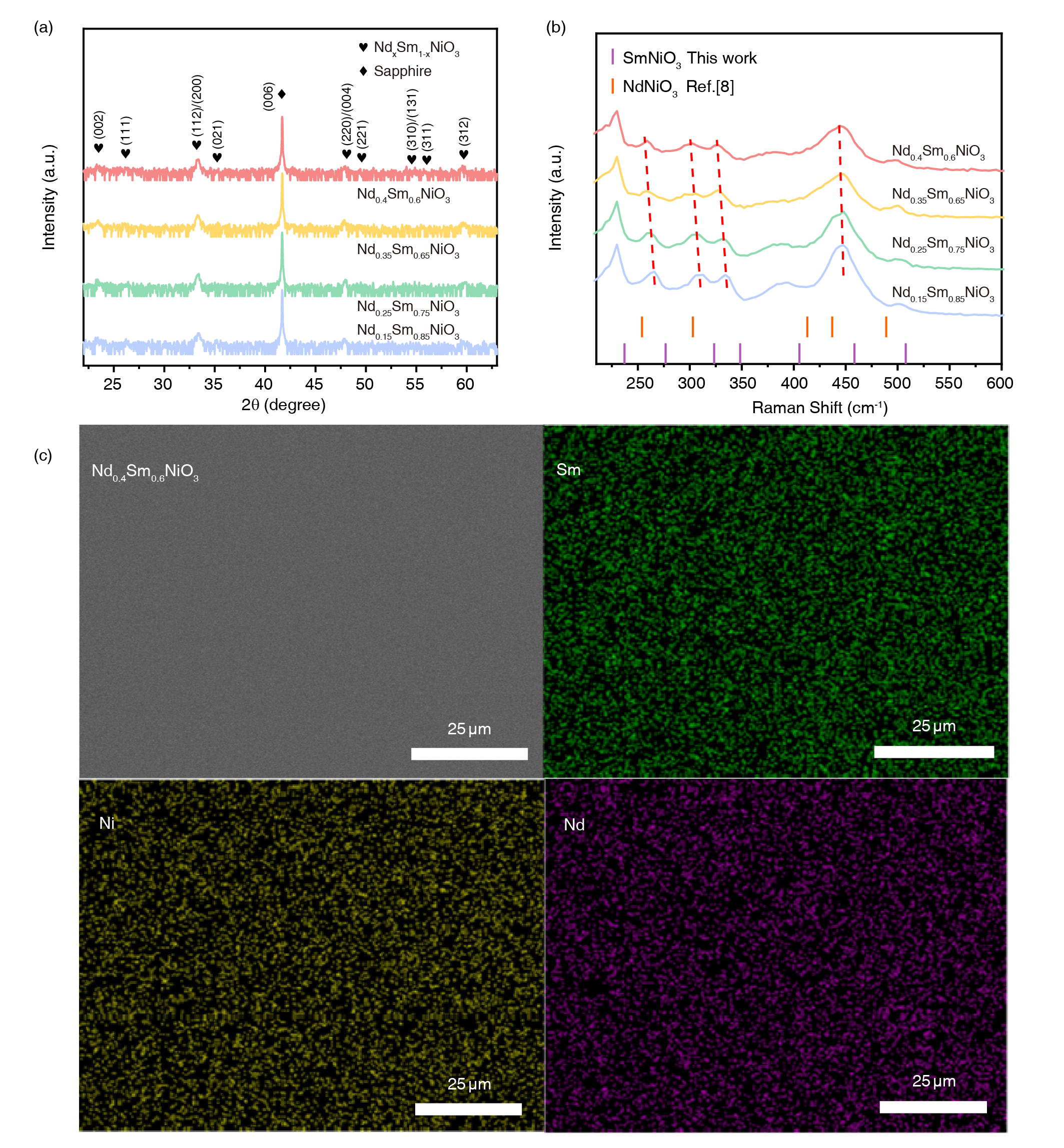


**Figure S12.** Structural characterization of Nd_x_Sm_1-x_NiO_3_ thin films. a) XRD results of Nd_x_Sm_1-x_NiO_3._ All the samples possess a perovskite structure similar to SmNiO_3._ No additional diffraction peaks appear, indicating the absence of secondary phases in the samples. b) Raman spectra of Nd_x_Sm_1-x_NiO_3_. With increasing the concentration of Nd, the Raman modes of Nd_x_Sm_1-x_NiO_3_ gradually shift to lower values and approach that of NdNiO_3_.^[8]^ c) Scanning electron microscopy (SEM) image and energy-dispersive spectroscopy (EDS) mapping of Nd_0.4_Sm_0.6_NiO_3_, where the Sm, Ni, and Nd elements are uniformly distributed.

**Table S1.** Summary of switchable optical devices based on infrared active materials.

| **Device** | **Method** | **Wavelength (μm)** | **ΔEmissivity** | **Feature** | **Ref.** |
| --- | --- | --- | --- | --- | --- |
| **Redox** | | | | | |
| Glass/ITO/NiO  /LiTaO_3_/Li_4_Ti_5_O_12_  /WO_3_/ITO | Electrical | 8-14 | 0.36 | All-solid-state device,  excellent cycle performance,  weak tunability | [9] |
| Glass/ITO/NiO_x_  /LiTaO_3_/WO_3_/ITO | Electrical | 8-14 | 0.37 |  | [10] |
| ITO/WO_3_/Au | Electrical | 2.5-25 | 0.32 |  | [11] |
| **Phase transition** | | | | | |
| VO_2_/Ge/Al/Si | Thermal | 8-14 | 0.38 | Planar structure, excellent thermal management performance, thermal hysteresis, modulation limited by the phase transition temperature | [12] |
| VO_2_/Al | Thermal | 10 | 0.56 |  | [13] |
| ITO/H_x_WO_3_/  Ta_2_O_5_/Pd/Mg_3_Ni | Electrical | 7.5-14 | 0.47 | Moderate tunability, weak stability | [14] |
| Si/Al/Ge_2_Sb_2_Te_5_ | Laser | 8-13 | 0.62 | High tunability, simple structure, micro-area modulation, high driven energy for phase change | [15] |
| La_0.825_Sr_0.175_MnO_3_  /BaF_2_/Al Multilayer | Thermal | 9.8 | 0.65 | Planar structure, high tunability, transition occurs lower than room temperature | [16] |
| La_0.7_Sr_0.3_MnO_3_  /Al/Quartz | Thermal | 2.5-25 | 0.43 |  | [17] |
| SmNiO_3_/Dielectric Multilayer/Al | Thermal | 5-16 | 0.42-0.45 | Simulation results, metamaterial moderate emissivity tunability | [18] |
| SmNiO_3_/Sapphire  /Pt | Electrical | 4-14.5 | 0.4 | Simulation results, planar structure, moderate emissivity tunability | [6] |
| SmNiO_3_/Si_3_N_4_/Si | Electrical | 2.5-8 | 0.4 | Planar structure, weak emissivity tunability at 7-14 μm | [6] |
| SmNiO_3_/Ge/Au Metasurface | Thermal | 8-14 | 0.367 | Complex preparation, weak emissivity tunability | [19] |
| SmNiO_3_/Sapphire | Electrical | 7-14 | 0.623 | Planar and simple structure, high emissivity tunability, perfect absorption | This work |
| **Conductive polymer** | | | | | |
| MWCNTs/PANI  /HMIM[TFSI] | Electrical | 8-14 | 0.23 | Easy preparation,  flexible devices, weak tunability | [20] |
| PVSK-PANI | Electrical | 8-14 | 0.46 |  | [21] |
| **Energy band engineering** | | | | | |
| Ion Gel/Graphene  /PET | Electrical | 7.5-14 | 0.27 | Excellent band compatibility, easy-to-modify optical band structure, complex preparation, moderate tunability | [22] |
| Cu/Separator  /Ion Gel/Graphene | Electrical | 7.5-14 | 0.55 |  | [23] |

References

[1] A. Shahsafi, P. Roney, Y. Zhou, Z. Zhang, Y. Z. Xiao, C. H. Wan, R. Wambold, J. Salman, Z. N. Yu, J. R. Li, J. T. Sadowski, R. Comin, S. Ramanathan M. A. Kats, *Proc. Natl. Acad. Sci. U. S. A.* **2019**, 116, 26402.

[2] M. K. Shi, M. M. Shen, X. Y. Guo, X. X. Jin, Y. X. Cao, Y. Y. Yang, W. J. Wang J. F. Wang, *ACS Nano.* **2021**, 15, 11396.

[3] M. A. Kats, D. Sharma, J. Lin, P. Genevet, R. Blanchard, Z. Yang, M. M. Qazilbash, D. N. Basov, S. Ramanathan F. Capasso, *Appl. Phys. Lett.* **2012**, 101, 221101.

[4] E. D. Palik, *Handbook of optical constants of solids II*, Academic Press, **1997**.

[5] J. Rensberg, S. Zhang, Y. Zhou, A. S. McLeod, C. Schwarz, M. Goldflam, M. K. Liu, J. Kerbusch, R. Nawrodt, S. Ramanathan, D. N. Basov, F. Capasso, C. Ronning M. A. Kats, *Nano Lett.* **2016**, 16, 1050.

[6] Z. Y. Li, Y. Zhou, H. Qi, Q. W. Pan, Z. Zhang, N. N. Shi, M. Lu, A. Stein, C. Y. Li, S. Ramanathan N. F. Yu, *Adv. Mater.* **2016**, 28, 9117.

[7] G. Catalan, *Phase Transit.* **2008**, 81, 729.

[8] M. Zaghrioui, A. Bulou, P. Laffez P. Lacorre, *J. Magn. Magn. Mater.* **2000**, 211, 238.

[9] Y. J. Xiao, X. Zhang, Z. T. Li, M. J. Chen, W. H. Sun, J. B. Deng J. P. Zhao, *Sol. Energy Mater. Sol. Cells.* **2024**, 268, 112735.

[10] X. Zhang, Y. L. Tian, W. J. Li, S. L. Dou, L. B. Wang, H. Y. Qu, J. P. Zhao Y. Li, *Sol. Energy Mater. Sol. Cells.* **2019**, 200, 109916.

[11] Z. T. Li, X. Zhang, B. Sun, H. L. Zhang, Y. Y. Fang, Y. J. Xiao, W. H. Sun, M. J. Chen, J. B. Deng, D. K. Yan Y. Li, *ACS Appl. Nano Mater.* **2024**, 7, 10625.

[12] J. K. Huang, L. M. Yuan, J. M. Liao, Y. T. Wang, Y. Liu, C. Ji, C. Huang X. G. Luo, *Adv. Mater. Technol.* **2024**, 9, 2400522.

[13] F. V. Ramirez-Cuevas, K. L. Gurunatha, L. Li, U. Zulfiqar, S. Sathasivam, M. K. Tiwari, I. P. Parkin I. Papakonstantinou, *Nat. Commun.* **2024**, 15, 9109.

[14] Y. J. Song, B. Z. Cheng, H. F. Cheng, Z. Meng D. Q. Liu, *ACS Appl. Mater. Interfaces.* **2024**, 16, 35372.

[15] D. Kang, Y. Kim M. Lee, *ACS Appl. Mater. Interfaces.* **2024**, 16, 4925.

[16] X. Z. Wang, M. J. Chen, H. F. Guo, X. Han, S. T. Lu, Y. Li X. H. Wu, *Appl. Therm. Eng.* **2024**, 257, 124197.

[17] D. S. Fan, Q. Li P. Dai, *Acta Astronaut.* **2016**, 121, 144.

[18] W. Hua, J. Li, Y. Du, H. Wang H. Zhou, *Optik (Netherlands).* **2024**, 296, 171556.

[19] B. Khalichi, A. Ghobadi, A. K. Osgouei, Z. R. Omam, H. Kocer E. Ozbay, *Nanoscale.* **2023**, 15, 10783.

[20] J. L. Gao, J. L. Zhou, M. Yuan, S. L. Yu, W. J. Ma, Z. X. Hu, H. X. Xiang M. F. Zhu, *ACS Appl. Mater. Interfaces.* **2024**, 16, 30421.

[21] S. W. Tang, J. L. Niu, C. L. Gu, Y. L. Liu, R. Z. Zheng, X. L. Weng C. Y. Jia, *Electrochim. Acta.* **2024**, 498, 144682.

[22] M. Lim, H. D. Kim, H. C. Shim, K. S. Kim, B. S. An J. H. Kim, *Nano Energy.* **2024**, 131, 110189.

[23] Y. F. Zhang, H. B. Ke, J. X. Li, Z. K. Weng, T. Lin, W. L. Peng, M. Y. Dai, R. Mu X. A. Zhang, *Appl. Phys. Lett.* **2022**, 120, 243504.
